# Omics approaches in understanding the benefits of plant-microbe interactions

**DOI:** 10.3389/fmicb.2024.1391059

**Published:** 2024-05-27

**Authors:** Archana Jain, Surendra Sarsaiya, Ranjan Singh, Qihai Gong, Qin Wu, Jingshan Shi

**Affiliations:** ^1^Key Laboratory of Basic Pharmacology and Joint International Research Laboratory of Ethnomedicine of Ministry of Education, Zunyi Medical University, Zunyi, China; ^2^Bioresource Institute for Healthy Utilization, Zunyi Medical University, Zunyi, China; ^3^Department of Microbiology, Faculty of Science, Dr. Rammanohar Lohia Avadh University, Ayodhya, Uttar Pradesh, India

**Keywords:** plant-microbe interactions, omics technologies, genomics, transcriptomics, proteomics, metabolomics

## Abstract

Plant-microbe interactions are pivotal for ecosystem dynamics and sustainable agriculture, and are influenced by various factors, such as host characteristics, environmental conditions, and human activities. Omics technologies, including genomics, transcriptomics, proteomics, and metabolomics, have revolutionized our understanding of these interactions. Genomics elucidates key genes, transcriptomics reveals gene expression dynamics, proteomics identifies essential proteins, and metabolomics profiles small molecules, thereby offering a holistic perspective. This review synthesizes diverse microbial-plant interactions, showcasing the application of omics in understanding mechanisms, such as nitrogen fixation, systemic resistance induction, mycorrhizal association, and pathogen-host interactions. Despite the challenges of data integration and ethical considerations, omics approaches promise advancements in precision intervention and resilient agricultural practices. Future research should address data integration challenges, enhance omics technology resolution, explore epigenomics, and understand plant-microbe dynamics under diverse conditions. In conclusion, omics technologies hold immense promise for optimizing agricultural strategies and fortifying resilient plant-microbe alliances, paving the way for sustainable agriculture and environmental stewardship.

## 1 Introduction

The profound effects of climate change, including shifting precipitation patterns, rising temperatures, and extreme weather, pose significant threats to agricultural practices and food security (Olanrewaju et al., 2024). Agriculture serves as a linchpin for economic growth, laying the foundation for secondary and tertiary industries (Shah et al., 2024). With global food production losses estimated at 352 million tons by 2070 owing to population growth (Sartori et al., 2024), there is an urgent need to accelerate food production over the next 30 years (Kimotho and Maina, 2024). Although crop improvement programs are underway, insufficient attention has been paid to modern techniques and balanced fertilization, leading to nutritional insecurity in staple crops (Jalal et al., 2024). The World Health Organization underscores the importance of food safety and urging measures to prevent foodborne diseases across various stages of food processing, production, storage, transportation, and consumption (Su et al., 2024). Sustainability of these challenges is crucial for global food systems, environmental stability, and climate resilience.

Recent advancements in omics approaches offer a promising avenue to address the challenges facing crop productivity by elucidating the benefits of plant-microbe interactions (Olanrewaju et al., 2024). Breakthroughs in analytical methodologies have provided comprehensive insights into the intricate dynamics of these interactions (Sharma et al., 2024). Omics techniques, including genomics, transcriptomics, and metabolomics, have proven invaluable in exploring the biochemical, physiological, and molecular aspects of plant-microbe interactions across various conditions (Tiwari et al., 2024). The integration of multi-omics data from different databases is essential for effective utilization of omics technologies, offering a comprehensive understanding of biological processes and interactions (Chao et al., 2024). These advancements have resulted in the accumulation of vast amounts of information at all levels, enabling deep insight into mechanisms under stressful conditions (Ahmed et al., 2024). The integrated use of multi-omics approaches enhances data analysis, visualization, and interpretation, facilitating a deeper understanding of biological processes.

The intricate relationship between plants and microbes plays a vital role in ecosystem dynamics and sustainable agriculture and is influenced by host-related elements, edaphic conditions, environmental dynamics, and anthropogenic factors (Rane et al., 2022). Understanding these factors is essential for unraveling the complexity of symbiotic relationships. Omics approaches, including genomics, transcriptomics, proteomics, and metabolomics, illuminate the molecular intricacies of plant-microbe symbiosis (Sarim et al., 2020). Genomics identifies key genes, transcriptomics reveals gene expression dynamics, proteomics identifies essential proteins, and metabolomics profiles small molecules, thereby offering a comprehensive perspective. Multi-omics techniques enable the study of various interactions, including obligate symbiotic relationships, such as those involving arbuscular mycorrhizal fungi and plants, extending to interactions with non-mycorrhizal microbes such as biofilms (Mishra et al., 2022). These insights underscore the transformative potential of omics technologies in advancing sustainable agriculture through a deeper understanding of beneficial plant-microbe relationships.

The integration of multi-omics and bioinformatics has revealed that plant-microbe interactions in polluted rhizospheres trigger the release of antioxidants and phytohormones, thereby activating plant defense mechanisms (Sengupta and Pal, 2021). Phytohormones serve as vital messengers that regulate plant growth and synthesize secondary metabolites (De Palma et al., 2022). Omics tools such as metagenomics and metatranscriptomics aid in the detection and analysis of phytohormones, deepening our understanding of their roles in these interactions. Understanding secretory metabolites can help in the design of next-generation microbial inoculants to enhance plant growth (Mishra et al., 2022). Advancements in sequencing technologies have facilitated the analysis of trace chemical substances, aiding the investigation of allelochemical biosynthesis and plant responses, thereby enhancing our understanding of rhizosphere chemistry (Weidenhamer et al., 2023). *Penicillium* and *Aspergillus* release phosphorus and potassium during mineral degradation, benefiting plant growth and defense in beneficial plant-microbe interactions (Paul et al., 2024). The presence of CAZymes from the GT4 and GT2 families suggests potential tripartite symbiotic relationships among plants, rhizospheric bacteriomes, and fungiomes, thereby guiding the application of omics tools to enhance plant resilience to environmental stress (Alshareef, 2024). *Salvia miltiorrhiza* boosts stress resistance through signals transmitted via AM (arbuscular mycorrhiza) hyphal networks post-pathogen stress from *Fusarium solani*, with metagenomics tools aiding in understanding and enhancing its resilience (Han et al., 2023).

There are limitations to each omics strategy that may affect the sensitivity or specificity of the technique. However, some of these limitations can be overcome by integrating different approaches. The objective of this review article is to explore the transformative impact of omics technologies on understanding plant-microbe interactions, which play a pivotal role in shaping ecosystems and promoting sustainable agriculture. This review explores the complexities of these interactions, which are influenced by various factors, such as host-related traits, environmental conditions, and anthropogenic activities. This highlights the emergence of omics approaches, including genomics, transcriptomics, proteomics, metabolomics, and epigenomics, which elucidate their roles in deciphering the molecular mechanisms underlying beneficial plant-microbe associations. By synthesizing findings from diverse microbial-plant interactions, this review showcases the application of omics technologies in unraveling mechanisms, such as nitrogen fixation, induction of systemic resistance, mycorrhizal association, and pathogen-host interactions. Furthermore, we discuss modern web-based omics tools that facilitate data analysis and interpretation. Ultimately, the review underscores the potential of omics approaches to revolutionize agricultural practices by offering insights into crop improvement, disease management, and sustainable agriculture, while addressing challenges in data integration and ethical considerations.

## 2 Molecular mechanisms underlying plant-microbe interactions

**Figure 1:**
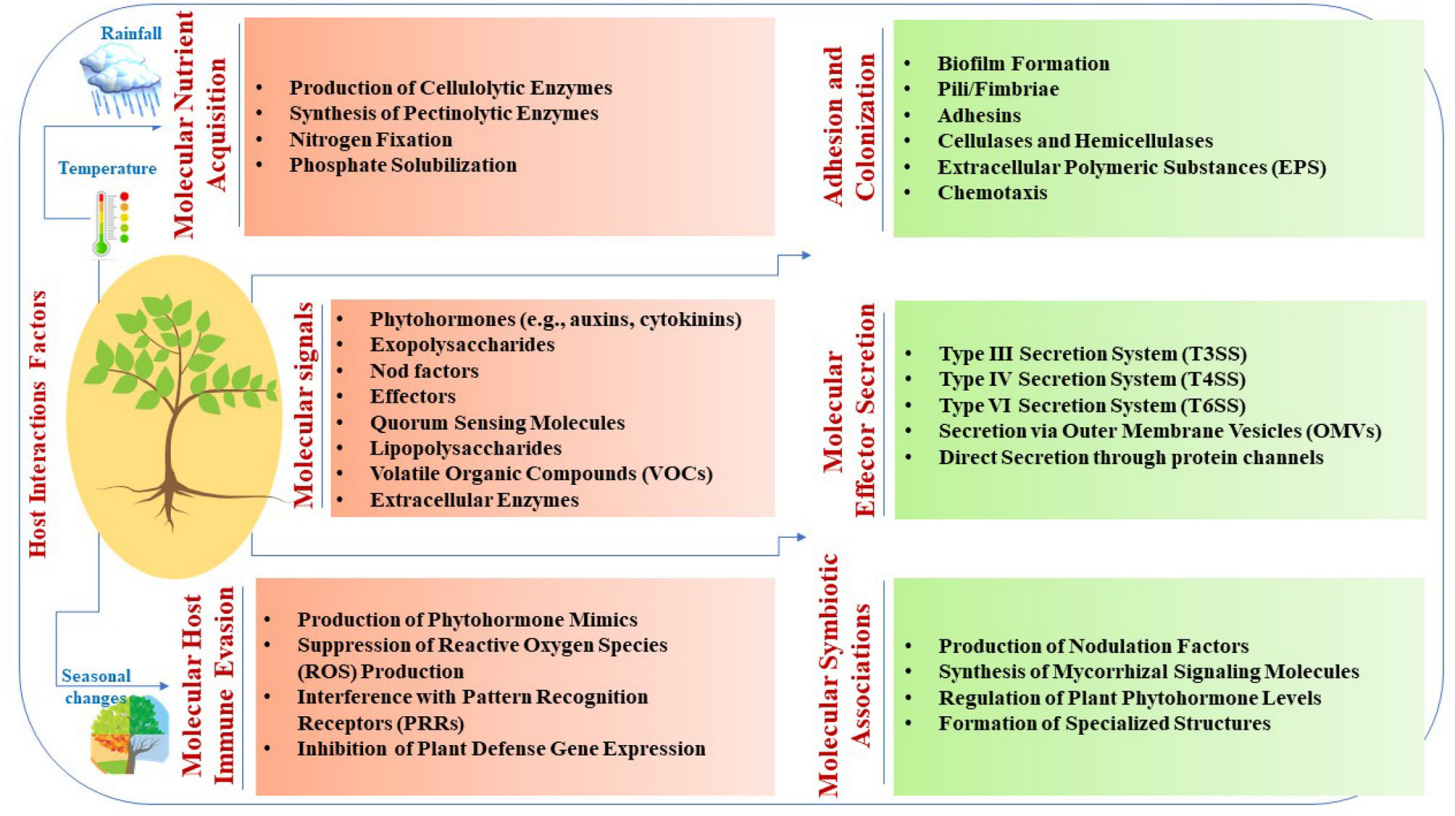
Molecular mechanisms underlying plant-microbe interactions.

Microbial interactions with host plants involve intricate molecular signaling mechanisms that are crucial for symbiosis, pathogenesis, and environmental adaptation (Table 1 and Figure 1). Phytohormones, such as auxins and cytokinins, produced by microbes such as *Rhizobium leguminosarum* stimulate plant growth and nutrient uptake through signaling by plant receptors (Meena et al., 2023). Exopolysaccharides synthesized by microbes such as *Pseudomonas fluorescens* facilitate attachment to plant surfaces, biofilm formation, and protection against environmental stress (Niazi et al., 2023). Nod factors secreted by rhizobia such as *Bradyrhizobium japonicum* induce nodulation signaling cascades and establish symbiotic relationships in leguminous plants (Grundy et al., 2023; Yuan et al., 2023). Effectors from pathogens, such as *Pseudomonas syringae*, manipulate host cellular processes to modulate plant immunity and facilitate infection (Kalita et al., 2024). Quorum-sensing molecules produced by microbes such as *Agrobacterium tumefaciens* induce systemic plant responses, whereas lipopolysaccharides from bacteria such as *Pseudomonas aeruginosa* trigger plant defense mechanisms (Majdura et al., 2023; Orozco-Mosqueda et al., 2023; Zhou et al., 2023; Elfaky, 2024). Volatile organic compounds produced by microbes such as *Trichoderma harzianum* also elicit plant systemic responses (Contreras-Cornejo et al., 2024). Furthermore, extracellular enzymes such as *Fusarium oxysporum* facilitate nutrient acquisition and microbial colonization (Niazi et al., 2023). These molecular mechanisms play pivotal roles in microbial interactions with host plants, influencing symbiosis, pathogenesis, and plant-microbe-environment interactions.

**Table 1:** Microbial-mediated molecular mechanisms of interactions with host plants.

**Molecular aspects**	**Mechanism steps**	**Microbial group**	**Interacting molecules**	**Interaction routes**	**Key function**	**References**
**Molecular signals**						
Phytohormones (e.g., auxins, cytokinins)	1. Production of phytohormones by microbes. 2. Secretion into the plant's rhizosphere. 3. Uptake and signaling by plant receptors.	*Rhizobium leguminosarum*	Plant receptors, hormone biosynthesis	Root exudates, rhizosphere colonization.	Stimulating plant growth, root development, and nutrient uptake.	Gómez-Godínez et al., 2023; Meena et al., 2023
Exopolysaccharides	1. Synthesis and secretion of EPS by microbes. 2. Adherence to plant surfaces. 3. Formation of biofilms.	*Pseudomonas fluorescens*	Plant cell surface, immune response	Root colonization, leaf surface.	Facilitating microbial attachment, biofilm formation, and protection against environmental stresses.	Bhattacharyya et al., 2023; Niazi et al., 2023
Nod factors	1. Production and secretion of Nod factors by *Rhizobia*. 2. Recognition by plant receptors (e.g., Nod-factor receptors). 3. Initiation of nodulation signaling cascade.	*Bradyrhizobium japonicum*	Plant receptor kinases	Rhizosphere, root colonization.	Inducing nodulation, establishment of symbiotic relationship in leguminous plants.	Grundy et al., 2023; Yuan et al., 2023
Effectors	1. Secretion of effector proteins by microbes. 2. Translocation into plant cells. 3. Manipulation of host cellular processes.	*Pseudomonas syringae*	Plant proteins, DNA	Direct injection, cell entry.	Modulating plant immunity, facilitating infection or establishing symbiosis.	Chiquito-Contreras et al., 2024; Kalita et al., 2024
Quorum sensing (QS) molecules	1. Production and release of QS molecules by microbes. 2. Diffusion through the environment. 3. Perception by plant receptors.	*Agrobacterium tumefaciens*	Microbial receptors, gene expression	Airborne, soilborne.	Inducing plant systemic responses, such as defense activation or stress tolerance.	Majdura et al., 2023; Elfaky, 2024
Lipopolysaccharides (LPS)	1. Release of LPS by Gram-negative bacteria. 2. Recognition by plant pattern recognition receptors (PRRs). 3. Activation of immune responses.	*Pseudomonas aeruginosa*	Plant pattern recognition receptors	Rhizosphere, root colonization.	Triggering plant defense mechanisms, such as pattern-triggered immunity (PTI).	Orozco-Mosqueda et al., 2023; Zhou et al., 2023
Volatile organic compounds (VOCs)	1. Production and release of VOCs by microbes. 2. Diffusion through air or soil. 3. Perception by plant receptors.	*Trichoderma harzianum*	Plant receptors, microbial communities	Airborne, soilborne.	Inducing plant systemic responses, such as defense activation or stress tolerance.	Ahlawat et al., 2024; Contreras-Cornejo et al., 2024
Extracellular enzymes	1. Secretion of enzymes by microbes. 2. Breakdown of complex molecules in the plant environment. 3. Utilization of resulting nutrients.	*Fusarium oxysporum*	Plant cell wall components, nutrients	Rhizosphere, leaf surface.	Facilitating nutrient acquisition, promoting microbial colonization.	Ajijah et al., 2023; Niazi et al., 2023
**Adhesion and colonization**						
Biofilm formation	1. Initial attachment of microbes to plant surfaces. 2. Production and secretion of extracellular polymeric substances (EPS). 3. Formation of microcolonies. 4. Maturation into a complex biofilm structure.	*Pseudomonas aeruginosa*	Extracellular matrix, Plant surfaces	Root surfaces, leaf surfaces, rhizosphere	Facilitating microbial attachment, providing protection against environmental stresses, enhancing nutrient availability, promoting interactions with host plants and other microorganisms.	Ajijah et al., 2023; Niazi et al., 2023
Pili/fimbriae	1. Production of pili/fimbriae by microbes. 2. Binding of pili/fimbriae to specific receptors on plant surfaces. 3. Tight adherence of microbes to plant cells.	*Escherichia coli*	Host cell receptors	Root surfaces, leaf surfaces	Facilitating strong attachment to plant tissues, promoting biofilm formation, mediating interactions with host plants, aiding in nutrient acquisition.	Bhattacharyya et al., 2023; Niazi et al., 2023
Adhesins	1. Production and secretion of adhesins by microbes. 2. Binding of adhesins to specific receptors on plant surfaces. 3. Establishment of stable attachment between microbes and plant cells.	*Agrobacterium tumefaciens*	Plant cell receptors, Extracellular matrix components	Root surfaces, leaf surfaces	Mediating specific and strong attachment to host plant tissues, facilitating colonization, biofilm formation, and establishment of symbiotic or pathogenic interactions.	Carezzano et al., 2023; Gupta et al., 2024
Cellulases and hemicellulases	1. Secretion of cellulases and hemicellulases by microbes. 2. Hydrolysis of cellulose and hemicellulose components in plant cell walls. 3. Facilitation of penetration into plant tissues.	*Fusarium oxysporum*	Plant cell wall components	Rhizosphere, root surfaces	Assisting in degradation of plant cell wall components, promoting invasion and colonization of plant tissues, aiding in nutrient acquisition and host tissue maceration.	Dutta et al., 2023
Extracellular polymeric substances (EPS)	1. Synthesis and secretion of EPS by microbes. 2. Accumulation of EPS around microbial cells and on plant surfaces. 3. Formation of a protective matrix for microbial communities.	*Bacillus subtilis*	Secreted polysaccharides, proteins, DNA	Root surfaces, leaf surfaces	Providing structural integrity to biofilms, protecting microbial cells from environmental stresses (e.g., desiccation, predation), facilitating nutrient retention and exchange, promoting adherence to surfaces and intercellular communication within microbial communities.	Ajijah et al., 2023; Santra and Banerjee, 2024
Chemotaxis	1. Detection of chemical gradients by microbial cells. 2. Movement of microbes toward favorable chemical signals (e.g., root exudates). 3. Directed migration and colonization of plant surfaces.	*Rhizobium leguminosarum*	Root exudates, wounds	Rhizosphere, root surfaces, leaf surfaces	Guiding microbial movement toward nutrient-rich or hospitable microenvironments, facilitating efficient colonization of plant tissues, enhancing interactions with host plants by responding to chemical cues.	Dunn and Becerra-Rivera, 2023; Dhiman et al., 2024
**Molecular effector secretion**						
Type III secretion system (T3SS)	1. Recognition of host cell by T3SS. 2. Activation of T3SS machinery. 3. Injection of effector proteins directly into host cell cytoplasm via a needle-like structure.	*Pseudomonas syringae*	Effector proteins, host cell receptors	Direct contact with host cells	Facilitating the delivery of effector proteins directly into host cells, manipulating host cellular processes, suppressing plant defenses, promoting pathogen virulence or symbiotic interactions.	De Ryck et al., 2023; Lee et al., 2023
Type IV secretion system (T4SS)	1. Assembly of T4SS machinery. 2. Formation of a protein channel connecting microbial and host cells. 3. Translocation of effector proteins from microbe to host cell cytoplasm.	*Agrobacterium tumefaciens*	Effector proteins, host cell cytoplasm	Direct contact with host cells	Mediating the transfer of effector proteins from microbial cells to host cells, facilitating manipulation of host cellular processes, promoting symbiotic interactions or pathogenesis.	Wangthaisong et al., 2023; Gupta et al., 2024
Type VI secretion system (T6SS)	1. Assembly of T6SS machinery. 2. Contact-dependent injection of effector proteins directly into adjacent microbial or host cells.	*Rhizobium etli*	Effector proteins, host cell proteins	Direct contact with host cells or other microbial cells	Participating in intercellular competition, modulating microbial community composition, facilitating host colonization or pathogenesis through the delivery of effectors into target cells.	De Sousa et al., 2023; Yin et al., 2024
Secretion via outer membrane vesicles (OMVs)	1. Budding of OMVs from microbial outer membrane. 2. Packaging of effector proteins into OMVs during biogenesis. 3. Release of OMVs into extracellular environment. 4. Uptake of OMVs by host cells or other microbes, leading to effector delivery.	*Escherichia coli*	Effector-containing vesicles, host cell membranes	Extracellular environment	Mediating long-distance delivery of effector proteins, facilitating communication between microbial cells, promoting interactions with host cells by delivering effectors or modulating host immune responses.	Chalupowicz et al., 2023; Pandey et al., 2024
Direct secretion through protein channels	1. Utilization of specialized protein channels (e.g., porins) for direct secretion of effector proteins from microbial cells into the extracellular environment or into host cells.	*Xanthomonas campestris*	Effector proteins, extracellular space	Extracellular environment or direct contact with host cells	Facilitating rapid secretion of effector proteins, modulating host cellular processes or immune responses, promoting microbial colonization or pathogenesis by directly interacting with host cells or affecting surrounding environment.	Maphosa et al., 2023
**Molecular nutrient acquisition**						
Production of cellulolytic enzymes	1. Expression and secretion of cellulolytic enzymes (e.g., cellulases) by microbes. 2. Hydrolysis of cellulose polymers into glucose monomers. 3. Uptake of glucose by microbes for energy and growth.	*Trichoderma reesei*	Cellulose in plant cell walls	Rhizosphere, root surfaces, plant debris	Facilitating the degradation of cellulose-rich plant materials, releasing nutrients for microbial utilization, enhancing soil fertility, and promoting interactions with plant roots.	Restrepo-Leal et al., 2023; Datta, 2024
Synthesis of pectinolytic enzymes	1. Production and secretion of pectinolytic enzymes (e.g., pectinases) by microbes. 2. Degradation of pectin, a component of plant cell walls, into simpler sugars and breakdown products. 3. Utilization of these products by microbes as carbon and energy sources.	*Pseudomonas syringae*	Pectin in plant cell walls	Rhizosphere, root surfaces	Facilitating the breakdown of pectin-rich plant tissues, promoting nutrient release, enhancing microbial growth and metabolism, and fostering interactions with host plants.	Anderson, 2023; Puranik et al., 2023
Nitrogen fixation	1. Expression and activity of nitrogenase enzymes by nitrogen-fixing bacteria (e.g., *Rhizobium, Bradyrhizobium*). 2. Conversion of atmospheric nitrogen (N_2_) into ammonia (NH_3_). 3. Incorporation of ammonia into organic molecules or its uptake by plants.	*Rhizobium leguminosarum*	Atmospheric nitrogen	Root nodules (in symbiotic associations), rhizosphere	Providing plants with accessible nitrogen in the form of ammonia, enhancing plant growth and productivity, improving soil fertility, and enabling sustainable agricultural practices.	Goyal and Habtewold, 2023; Mahapatra et al., 2023
Phosphate solubilization	1. Production and secretion of organic acids (e.g., citric acid, gluconic acid) by phosphate-solubilizing microbes. 2. Acidification of the rhizosphere environment. 3. Solubilization of insoluble phosphate minerals, releasing phosphate ions (${\text{PO}}_{4}^{3-}$) into the soil solution.	*Bacillus megaterium*	Phosphate in soil minerals	Rhizosphere, root surfaces	Enhancing the availability of phosphate for plant uptake, promoting plant growth and development, improving nutrient use efficiency, and contributing to sustainable agricultural practices.	da Silva et al., 2023; Pantigoso et al., 2023
**Molecular host immune evasion**						
Production of phytohormone mimics	1. Synthesis and secretion of compounds mimicking plant phytohormones (e.g., salicylic acid, jasmonic acid) by microbes. 2. Binding to plant hormone receptors. 3. Modulation of plant hormone signaling pathways.	*Pseudomonas syringae*	Plant hormone receptors	Rhizosphere, leaf surface	Disrupting plant hormonal balance, manipulating plant growth and defense responses, facilitating microbial colonization, and promoting pathogen virulence or symbiosis.	Ravelo-Ortega et al., 2023; Selwal et al., 2023
Suppression of reactive oxygen species (ROS) production	1. Secretion of effector proteins by microbes. 2. Interference with plant signaling pathways involved in ROS production (e.g., mitogen-activated protein kinase (MAPK) cascade). 3. Inhibition of ROS burst in response to microbial invasion or elicitation.	*Xanthomonas campestris*	Plant enzymes involved in ROS production	Rhizosphere, root surfaces, leaf surfaces	Preventing the activation of plant defense responses associated with ROS production, promoting microbial survival and proliferation, facilitating infection or establishment of symbiosis.	Gupta et al., 2023; Khoshru et al., 2023
Interference with pattern recognition receptors (PRRs)	1. Secretion of effector proteins or metabolites by microbes. 2. Binding to or modification of plant PRRs. 3. Disruption of PRR-mediated recognition of microbial-associated molecular patterns (MAMPs).	*Agrobacterium tumefaciens*	Plant pattern recognition receptors	Rhizosphere, root surfaces, leaf surfaces	Evading plant immune surveillance, avoiding recognition by the host immune system, suppressing the activation of downstream defense responses, and promoting microbial colonization or infection.	Barka et al., 2023; Jiang et al., 2023; Wu et al., 2024
Inhibition of plant defense gene expression	1. Production and secretion of effector proteins by microbes. 2. Translocation into plant cells. 3. Suppression or manipulation of transcriptional regulators or signaling components involved in plant defense gene expression.	*Fusarium oxysporum*	Plant transcription factors	Direct contact with host cells	Suppressing the expression of defense-related genes, dampening plant immune responses, promoting microbial colonization or infection, and facilitating establishment of symbiotic associations.	Manoharan et al., 2023; Rani et al., 2023
**Molecular symbiotic associations**						
Production of nodulation factors	1. Synthesis and secretion of nodulation factors (Nod factors) by *Rhizobia* bacteria. 2. Perception of Nod factors by plant receptors (e.g., Nod-factor receptors). 3. Initiation of signaling cascades leading to nodulation and symbiosis.	*Rhizobium leguminosarum*	Legume root receptors	Rhizosphere, root surfaces	Inducing nodulation in leguminous plants, initiating the formation of root nodules, facilitating the establishment of nitrogen-fixing symbiosis, and enhancing plant growth and nitrogen acquisition.	Grundy et al., 2023; Shumilina et al., 2023
Synthesis of mycorrhizal signaling molecules	1. Production and release of signaling molecules (e.g., strigolactones) by host plants in response to low phosphate availability. 2. Perception of signaling molecules by mycorrhizal fungi. 3. Initiation of mycorrhizal symbiosis and hyphal colonization.	*Glomus intraradices*	Plant root exudates, mycorrhizal fungi	Rhizosphere, root surfaces	Facilitating the establishment of mycorrhizal associations, promoting hyphal growth and colonization of plant roots, enhancing nutrient uptake (e.g., phosphorus), and improving plant growth and stress tolerance.	Wahab et al., 2023; Koshila Ravi and Muthukumar, 2024
Regulation of plant phytohormone levels	1. Secretion of microbial metabolites or effector proteins that modulate plant hormone signaling pathways. 2. Interference with plant phytohormone biosynthesis, perception, or signaling. 3. Manipulation of host physiological processes and development.	*Bradyrhizobium japonicum*	Plant hormone receptors	Rhizosphere, root surfaces, leaf surfaces	Modulating plant growth, development, and stress responses, optimizing resource allocation, enhancing plant fitness and stress tolerance, and promoting mutualistic interactions with microbes.	Gómez-Godínez et al., 2023; Kaya, 2024
Formation of specialized structures	1. Expression of microbial genes involved in the formation of specialized structures (e.g., nodules, arbuscules). 2. Induction of morphological changes in host tissues. 3. Development of symbiotic structures facilitating nutrient exchange and metabolic cooperation.	*Frankia* spp.	Plant root cells	Root surfaces, intracellular colonization	Facilitating nutrient exchange between microbes and host plants, enhancing nutrient acquisition and utilization, promoting mutualistic interactions, and improving plant growth and fitness.	Gasser et al., 2023; Scaria and Ravi, 2023

The adhesion and colonization of microbes on plant surfaces involve intricate molecular processes that are essential for establishing symbiotic or pathogenic relationships. Microbes utilize various strategies such as EPS (exopolysaccharides) production, pili/fimbriae binding, adhesin secretion, and enzymatic degradation of plant cell wall components (Bhattacharyya et al., 2023; Carezzano et al., 2023; Dutta et al., 2023; Niazi et al., 2023). Additionally, chemical gradients, such as root exudates, guide microbial migration and colonization of plant surfaces (Dunn and Becerra-Rivera, 2023; Dhiman et al., 2024). Molecular effector secretion by microbial pathogens involves sophisticated mechanisms for manipulating host cellular processes. Pathogens utilize various secretion systems, including T3SS, T4SS, T6SS, outer membrane vesicles (OMVs), and direct secretion systems to deliver effector proteins into host cells (Chalupowicz et al., 2023; De Ryck et al., 2023; De Sousa et al., 2023; Maphosa et al., 2023; Wangthaisong et al., 2023). These effectors manipulate the host cellular processes to promote infection and pathogenesis. The T4SS system in rhizobial species, akin to *Agrobacterium tumefaciens* Vir proteins, shares similarities in key components, such as trbD (virB3), trbI (virB10), and trbL (virB6). These elements facilitate genetic exchange through tra genes, such as traD, traR, and traG, which are analogous to virD4 and are essential for conjugative transfer within rhizobial populations. Additionally, *Mesorhizobium loti* strain R7A utilizes its T4SS to transfer specific proteins, such as Msi059 (a protease) and Msi061 (involved in ubiquitinylation), contributing to various cellular processes and interactions with host plants (Gupta et al., 2024). Furthermore, T6SS genes are prevalent in plant-associated bacteria, including rhizobial species, and play a crucial role in interbacterial competition, providing advantages in multimicrobial plant environments (De Sousa et al., 2023). Moreover, mutations in immune receptors, such as EFR, FLS2, and BAK1, minimally affect OMV-induced immune priming (Chalupowicz et al., 2023).

Microbial nutrient acquisition involves intricate mechanisms for acquiring essential nutrients from the plant environment. Microbes produce enzymes and metabolites that degrade complex plant molecules and enhance nutrient availability (Anderson, 2023; Puranik et al., 2023). Nitrogen-fixing bacteria such as *Rhizobium leguminosarum* convert atmospheric nitrogen to ammonia, thereby enhancing plant growth and soil fertility (Goyal and Habtewold, 2023). Phosphate-solubilizing microbes produce organic acids to solubilize phosphate minerals and promote plant growth (da Silva et al., 2023). Microbial immune evasion strategies involve sophisticated mechanisms for subverting plant defense responses. Pathogens mimic plant hormones, interfere with signaling pathways, and suppress immune surveillance to promote colonization and infection (Barka et al., 2023; Khoshru et al., 2023; Ravelo-Ortega et al., 2023). These mechanisms highlight the ability of microbes to circumvent plant immune defenses. Symbiotic associations involve intricate mechanisms that establish mutualistic relationships. Microbes synthesize signaling molecules, respond to host signals, regulate hormone pathways, and form specialized structures for nutrient exchange (Scaria and Ravi, 2023; Shumilina et al., 2023; Kaya, 2024; Koshila Ravi and Muthukumar, 2024). These mechanisms underscore the complexity of the symbiotic interactions between microbes and host plants. These symbiotic associations not only contribute to plant health and vigor but also foster ecosystem resilience and sustainability by enhancing soil fertility and nutrient cycling (Scaria and Ravi, 2023; Wahab et al., 2023). These examples highlight the complexity and versatility of the molecular mechanisms underlying plant-microbe interactions, underscoring the importance of understanding these processes for elucidating host-microbe dynamics and developing strategies for sustainable agriculture and disease management.

## 3 Omics approaches mechanism in beneficial plant-microbe interaction

**Figure 2:**
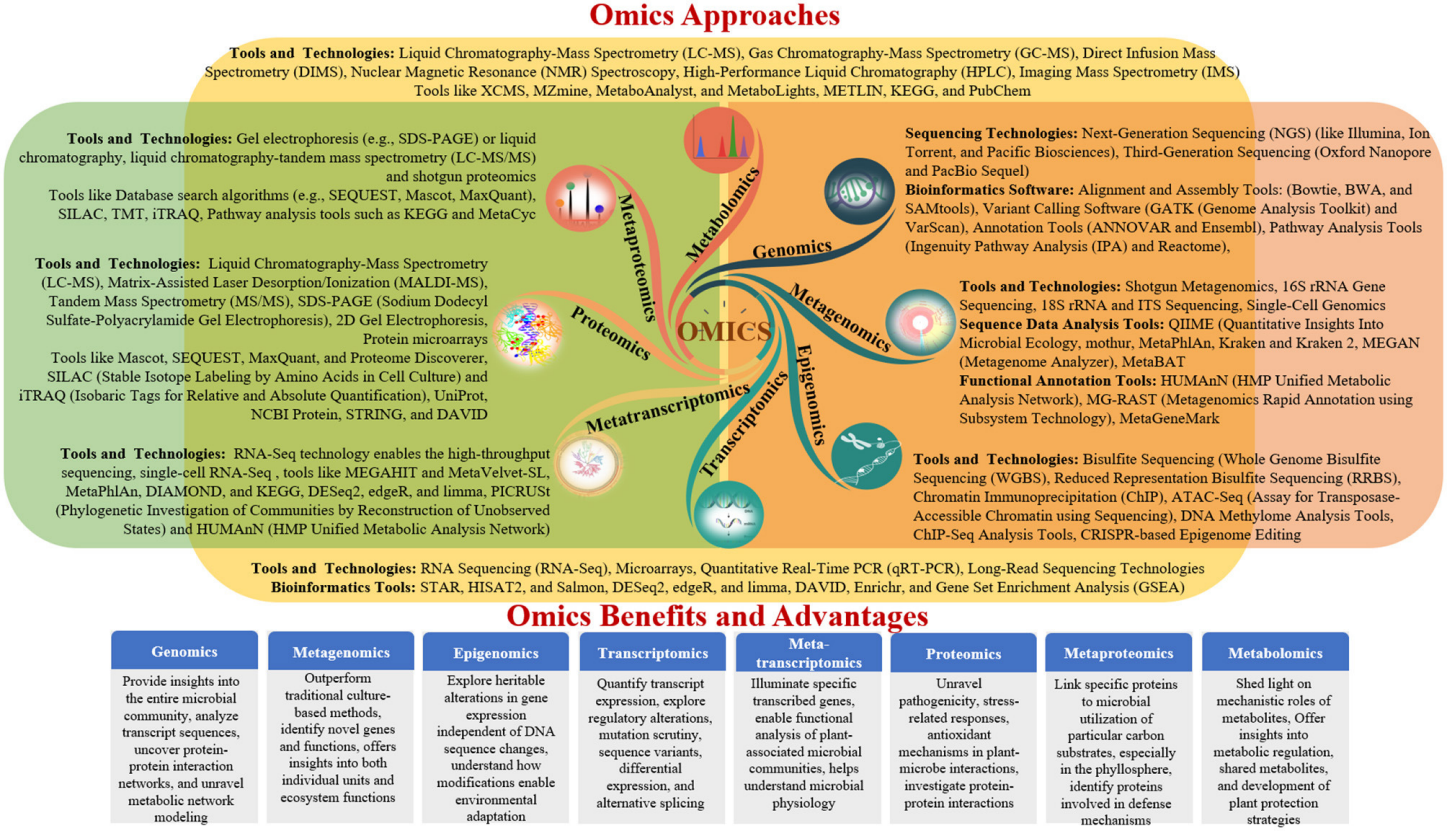
Diverse omics approaches for studying plant–microbe interactions.

Advances in molecular biology and high-resolution analytical technologies have enabled the comprehensive investigation of plant-microbe interactions through genomics, metagenomics, transcriptomics, proteomics, and metabolomics (Diwan et al., 2022). These omics approaches decipher the functional and structural aspects of genes, provide insights into the entire microbial community, analyse transcript sequences, uncover protein-protein interaction networks, and unravel metabolic network modeling (Zulfiqar et al., 2024). Together, these findings enhance our understanding of the effects of omics on interactions between plants and microbes. This review provides a comprehensive overview of each omics approach and its specific focus areas in elucidating beneficial plant-microbe interactions, ultimately contributing to the development of sustainable agricultural practices (Table 2 and Figure 2).

**Table 2:** Omics approaches for studying diverse plant-microbe interactions.

**Omics approach**	**Microbial examples**	**Function**	**Benefits**	**Microbial group**	**Advantages**	**References**
Genomics	*Klebsiella pneumoniae, Azotobacter vinelandii, Rhodospirillum rubrum*, and *Rhodobacter capsulatus*	Decipher structural and functional aspects of genes	Provide insights into the entire microbial community, analyze transcript sequences, uncover protein-protein interaction networks, and unravel metabolic network modeling	Symbiotic nitrogen-fixing bacteria	Reduced sequencing costs, accessibility of whole-genome sequences	Diwan et al., 2022
Metagenomics	*Rhizobium*-legume symbiosis, *Alternaria alternata*, Apple host response	Capture genetic sequence information across entire microbial communities	Outperform traditional culture-based methods, identify novel genes and functions	Symbiotic nitrogen-fixing bacteria	Offers insights into both individual units and ecosystem functions	Zulfiqar et al., 2024
Epigenomics	*Neobacillus* (Naxos tubers), Plant response to *Pseudomonas syringae*, Correlation of genomic and epigenomic traits in fungi	Analyze epigenetic modifications and genetic material changes in both plants and microbes	Explore heritable alterations in gene expression independent of DNA sequence changes, understand how modifications enable environmental adaptation, explore inheritance patterns and potential transgenerational effects	Mycorrhizal fungi, endophytes; plant growth-promoting rhizobacteria (PGPR), biocontrol agents	Explores changes in DNA methylation patterns, histone protein modifications, and non-coding RNAs	Samantara et al., 2021; Ali et al., 2023; Boutsika et al., 2023; Doddavarapu et al., 2024; Joubert and Krasileva, 2024; Masenya et al., 2024
Transcriptomics	*Pseudomonas fluorescens*-induced systemic resistance in *Arabidopsis, Penicillium expansum*-induced post-harvest fruit decay and mycotoxin production in blueberries, *Delftia acidovorans* with canola and soybean plant roots	Detect and quantify RNA molecules, linking gene function with specific conditions	Quantify transcript expression, explore regulatory alterations, mutation scrutiny, sequence variants, differential expression, and alternative splicing	Plant growth-promoting rhizobacteria (PGPR), biocontrol agents	Provides sequences of all transcripts in a sample, enabling exploration of molecular mechanisms	Assis et al., 2014; Chen et al., 2022; Gamalero et al., 2022; Jeon et al., 2022; Rathnasamy et al., 2023
Metatranscriptomics	*Xylella fastidiosa*-induced Pierce's disease in grapevine	Examine gene expression across the microbial community, revealing dynamic functions and providing insights into complex microbial physiology	Illuminate specific transcribed genes, enable functional analysis of plant-associated microbial communities	Biocontrol agents, Disease response	Characterizes real-time mRNA expression in environmental samples, helps understand microbial physiology	Dubey et al., 2020; Kumar et al., 2021; Gamalero et al., 2022; Saarenpää et al., 2023
Proteomics	Symbiont *S. meliloti* and phytopathogen *P. syringae*	Analyze expressed genes, revealing the role of proteins in microbial metabolic processes across diverse habitats	Unravel pathogenicity, stress-related responses, antioxidant mechanisms in plant-microbe interactions	Symbiotic interactions	Investigate protein-protein interactions, identify and study protein functions	Khatabi et al., 2019; Sarim et al., 2020; Jain et al., 2021; Chandok et al., 2022
Metaproteomics	*Trichoderma harzianum*-induced biocontrol	Study soil fertility, plant-microbe interactions, nutrient cycling, and bioremediation by identifying biocontrol-related proteins, pathogenicity factors, and host defense mechanisms	Link specific proteins to microbial utilization of particular carbon substrates, especially in the phyllosphere	Plant growth-promoting rhizobacteria (PGPR), biocontrol agents	Analyze bacterial communities, identify proteins involved in pathogenicity and host defense mechanisms	Priya et al., 2021; Rane et al., 2022
Metabolomics	Mycorrhizal association for nutrient uptake in *Glomus intraradices* and tomato	Investigate qualitative and quantitative insights into the symbiotic mechanisms of bacteria and fungi with plants	Shed light on mechanistic roles of metabolites in plant-microbe interactions	Mycorrhizal fungi, endophytes	Offer insights into metabolic regulation, shared metabolites, and development of plant protection strategies	Shafi et al., 2021; Chen et al., 2022; Manickam et al., 2023; Singh et al., 2023; Demiwal et al., 2024

### 3.1 Genomics

Recent advances in genomics have revealed that plants have adapted to a wide range of biotic interactions that extend beyond their relationships with beneficial symbionts (Carper et al., 2022; Chiquito-Contreras et al., 2024). Advancements in next-generation sequencing (NGS) technologies have not only reduced sequencing costs but also accelerated access to whole-genome sequences, *de novo* assemblies, and resequencing of multiple strains within species (Crandall et al., 2020). Through these analyses, researchers have uncovered the genetic mechanisms responsible for crucial functions such as nitrogen fixation and synthesis of growth-promoting compounds. Metagenomic approaches further reveal the diversity and functional potential of microbial communities, shedding light on their contribution to plant health. Transcriptomic and proteomic analyses complement genomic insights by elucidating gene expression and protein profiles during interactions, thereby revealing the molecular underpinnings. Leveraging this genomic knowledge, synthetic biology strategies enable the engineering of beneficial traits in plants and microbes, whereas microbiome engineering endeavors to optimize microbial communities to bolster plant vigor and productivity (Chandok et al., 2022).

#### 3.1.1 Metagenomics

Metagenomics systematically captures genetic sequence information across entire microbial communities, outperforming traditional culture-based methods (Ghosh et al., 2019). It encompasses structural and functional approaches, and offers insights into both individual units and ecosystem functions. Initially, using Sanger sequencing and later transitioning to next-generation sequencing, metagenomics employed 16S rRNA (ribosomal ribonucleic acid) and random shotgun sequencing to identify novel genes (Regalado et al., 2020). Metagenomics has been applied to diverse areas, from identifying novel nitrogen-fixing genes in *Rhizobium*-legume symbiosis (Regalado et al., 2020) to detecting mycotoxin biosynthetic genes in *Alternaria alternata* and its host response in apples (Bhargava et al., 2019). This involves sequencing of environmental models comprising various life forms, many of which are unculturable. The sequencing of universal genomic regions, such as ribosomal RNA genes, from diverse microbial species has proven successful (Zhang et al., 2021). Metagenomic analysis can be performed through amplicon targeting or shotgun sequencing to provide valuable insights into plant-associated microbial communities.

Nif operons, such as nifHDK, nifRLA, nifENB, nifUSVM, nifJ, and nifWF, constitute a core genetic component of diazotrophs, which are free-living anaerobic bacteria capable of nitrogen fixation. Notable examples include *Azotobacter vinelandii, Klebsiella pneumoniae, Rhodobacter capsulatus*, and *Rhodospirillum rubrum* (Idris Usman and Muazu Wali, 2024). These genes are pivotal in nitrogen fixation, as they synthesize essential components and regulate enzymes crucial for the process. Advancements in omics technologies, such as genomics and gene manipulation, have tremendous potential for bolstering crop yield. Additionally, the rhizosphere microbiome synthesizes ACC (1-aminocyclopropane-1-carboxylate) deaminase, which facilitates the breakdown of ACC into α-ketobutyrate and ammonia. This breakdown aids in plant nutrition and reduces the concentration of ethylene, thus mitigating its adverse effects. The production of ACC deaminase (ACCD) by plant growth-promoting rhizobacteria significantly enhances plant resilience to various abiotic stressors (Kumari and Kumawat, 2024). The ACCD structural gene (AcdS) is found in the genomes of rhizospheric bacteria, symbiotic rhizobia, and endophytes. In nitrogen-fixing bacteria, such as *Rhizobia* and *Mesorhizobium*, acdS expression is regulated differently. Specifically, the NifA2 gene and σ54 sigma factor control acdS expression in N-fixing bacteria (Larekeng et al., 2024). The genetic makeup of *Rhizobium* sp. and *Hydrogenophaga* sp. was comprehensively characterized using Illumina and Nanopore sequencers coupled with MaSuRCA assembly, revealing genes involved in metabolic functions and compound synthesis that contribute to plant growth stimulation. These findings underscore the symbiotic relationship between rhizobacteria and plants, potentially implicating processes such as nitrogen fixation and production of growth-promoting compounds (Ilangumaran et al., 2024). Notably, under non-sterile conditions, only *Pseudomonas sivasensis* exhibited notable promotion of canola growth, possibly because of the presence of additional genes in its genome, including those responsible for ACC deaminase (acdA), indole-3-acetic acid (IAA) production (trpF and trpG), and siderophore production (fbpA, mbtH, and acrB), which enhances its capacity to stimulate plant growth (Swiatczak et al., 2024). Moreover, rhizobacteria trigger defense responses through the expression of pathogenesis-related proteins (PR-proteins), such as chitinases, which are pivotal for defense mechanisms. A genome-wide examination of soybean chitinases identified GmChi01, GmChi02, and GmChi16, whose defense contributions were verified against *Fusarium oxysporum* in *Arabidopsis* transgenic lines, with GmChi02 and GmChi16 enhancing defense against *F. oxysporum*, whereas GmChi02 was significantly induced by *Burkholderia ambifaria* (Chen et al., 2024). Furthermore, transgenic expression of entomocidal and antimicrobial proteins from *Bacillus thuringiensis* (Bt) in maize is an alternative to host resistance against *Fusarium* ear rot (FER), and the recruitment of beneficial microbes is dependent on the genetic background of the host, highlighting the importance of microbe-microbe interactions in modulating FER severity (Adams et al., 2024).

#### 3.1.2 Epigenomics

Epigenomics, as a tool for studying plant-microbe interactions, involves a thorough analysis of epigenetic modifications and genetic material changes in both plants and microbes. This approach explores heritable alterations in gene expression independent of DNA (deoxyribonucleic acid) sequence changes (Samantara et al., 2021). Epigenomics in plant-microbe interactions involves studying changes in DNA methylation patterns, histone protein modifications such as acetylation and methylation, and the roles of non-coding RNAs such as microRNAs. It also includes analyzing chromatin remodeling, understanding how these modifications enable environmental adaptation, and exploring their inheritance patterns and potential transgenerational effects on plant and microbial traits (Ali et al., 2023). Furthermore, epigenetics plays a crucial role in plant responses to diseases, encompassing both biotic and abiotic stresses. EpiEffectors, such as zinc finger (ZF), transcription activator-like effector (TALE), or modified CRISPR/Cas9 complexes, such as dead Cas9 (dCas9), are used for targeted epigenome editing, and are often fused with the catalytic domain of epigenetic enzymes for precise modifications (Doddavarapu et al., 2024). Active DNA demethylation has been shown to positively affect plant resistance to pathogens such as *Pseudomonas syringae* (Masenya et al., 2024). The proline-alanine-valine (PAV) gene may be associated with specific genomic and epigenomic traits in fungi, offering potential predictive insights into fungal plant pathogen adaptation to hosts and aiding in the development of more effective disease prevention strategies (Joubert and Krasileva, 2024). Epigenomic analysis revealed that hypermethylation of xyloglucan endotransglycosylase protein could be influenced by *Neobacillus*, a dominant and highly abundant species found exclusively in Naxos tubers (Boutsika et al., 2023). This integrated approach identifies pivotal microbial taxa, their pathways, and epigenetic markers shaping host-microbe interactions, and offers novel insights into agriculture (Masenya et al., 2024).

### 3.2 Transcriptomics and metatranscriptomics

Transcriptomics employs next-generation sequencing (NGS) to detect and quantify RNA molecules in biological samples, linking gene functions under specific conditions. It uses methods such as microarray analysis, SOLiD-SAGE, and RNA sequencing (RNA-seq) for transcriptional profiling (Chen et al., 2022). Transcriptome analysis quantifies the expression and provides sequences of all samples, enabling the exploration of regulatory alterations, mutation scrutiny, sequence variants, differential expression, and alternative splicing (Weidemüller et al., 2021; Jeon et al., 2022). Transcriptome analyses have identified genes crucial in transitioning plant-microbe interactions from mutualistic to pathogenic, shedding light on disrupted associations (Rathnasamy et al., 2023). Assis et al. (2014) employed transcriptomics to uncover plant defense-related genes during *Pseudomonas fluorescens*-induced systemic resistance in *Arabidopsis*, while Jeon et al. (2022) utilized transcriptomics to unveil host pathways responding to pathogen infection in *Penicillium expansum*-induced post-harvest fruit decay and mycotoxin production in blueberries. RNA-seq transcriptomic analysis elucidated the mechanisms underlying the interactions of the plant growth-promoting bacteria (PGPB) *Delftia acidovorans* RAY209 with canola and soybean plant roots, focusing on the colonization process (Gamalero et al., 2022). Metatranscriptomics examines gene expression across the microbial community, revealing dynamic functions and providing insights into the complex microbial physiology (Dubey et al., 2020). It is a powerful tool for analyzing functional profiles and understanding the structure of microbial communities. Metatranscriptomics, which investigates total mRNA, provides real-time insights into mRNA expression in environmental samples (Dubey et al., 2020). This technique aids in identifying specific transcribed genes and enables the functional analysis of plant-associated microbial communities (Kumar et al., 2021; Gamalero et al., 2022). Comparative metatranscriptomic analyses of the microbial expression levels in uncontaminated and contaminated samples will enhance phytoremediation strategies. Dubey et al. (2020) utilized metatranscriptomics to examine transcriptome-wide changes in gene expression during *Xylella fastidiosa*-induced Pierce's disease in grapevines.

### 3.3 Proteomics and metaproteomics

Proteomic studies have analyzed expressed genes, revealing the role of proteins in microbial metabolic processes across diverse habitats. During plant-microbe interactions, proteins play a crucial role in cellular homeostasis, signaling networks, and defense responses. Proteomic analysis offers practical insights into these interactions, particularly in unculturable microbes. Khatabi et al. (2019) used proteomics to study pathogen effectors and host proteins in *Phytophthora infestans*-induced disease in potatoes, while Jain et al. (2021) employed proteomics to identify secreted proteins in *Erwinia amylovora*-induced fire blight disease in apples. Proteomic techniques are instrumental in unraveling the pathogenicity, stress-related responses, and antioxidant mechanisms involved in plant-microbe interactions, providing valuable insights into physiological and cellular processes. Functional proteomics utilizes the yeast two-hybrid system (Y2H) to investigate protein-protein interactions (PPIs) by employing “Prey” and “Bait” proteins. Mass spectrometric data analysis tools, such as PeptIdent, MultiIdent, MASCOT, SEQUEST, and Sherpa, facilitate proteomic analysis, enabling researchers to identify and study protein interactions and functions. Additionally, programs such as ProFound, MS-Fit, MOWSE, PepSea, PepFrag, and MS-Tag offer specialized analyses of MALDI-TOF and MS/MS spectra, enhancing the depth of proteomic investigation (Chandok et al., 2022). Gel-based resolution methods, such as two-dimensional gel electrophoresis (2DE) and differential gel electrophoresis (DIGE) coupled with mass spectrometry (MS), enable the identification and quantification of proteins. Studies have identified plant-related proteins using 2D-LC/MS/MS in *M. truncatula* nodules and revealed differential protein expression in rice tissues treated with *S. meliloti*. Additionally, comparative proteomic analyses have elucidated the protein secretion patterns of the symbiont *S. meliloti* and phytopathogen *P. syringae* DC3000 in response to root exudates from different host plants. Investigations into near-isogenic line alleles of Fhb1 have provided insights into *Fusarium graminearum* tolerance, while studies on beneficial microbes in the pea rhizosphere have highlighted differential proteomic responses upon infection by the necrotrophic fungus, *Sclerotinia sclerotiorum*. These findings underscore the importance of proteomic approaches in understanding the complex interactions between plants and microbes (Jain et al., 2021). Comparative proteomics of *the B. cinerea-*secreted proteome identified altered proteins, primarily pectinases, that are responsible for cell wall degradation. The secreted proteome also features a predominance of serine proteases, followed by metalloproteases, and threonine proteases (Sarim et al., 2020). iTRAQ proteomics revealed the regulatory mechanism in both resistant and susceptible rice cultivars against *Magnaporthe oryzae*. Metaproteomic investigations are pivotal for studying soil fertility, plant-microbe interactions, nutrient cycling, and bioremediation. Priya et al. (2021) conducted the first metaproteogenomic study analyzing bacterial communities in the phyllosphere of various plants, revealing consistency in dominant bacterial taxa and identified proteins across different species. Rane et al. (2022) used metaproteomics to identify biocontrol-related proteins in *Trichoderma harzianum*-induced biocontrol, whereas Priya et al. (2021) employed metaproteomics to identify proteins involved in the pathogenicity of *Fusarium oxysporum*-induced vascular wilt and host defense modulation in watermelon. Metaproteomics can link specific proteins to microbial utilization of particular carbon substrates, especially in the phyllosphere.

### 3.4 Metabolomics

Over the past decade, metabolomics has emerged as a well-established technique for investigating plant-microbe interactions, providing both qualitative and quantitative insights into the symbiotic mechanisms of bacteria and fungi with plants (Chouchani, 2022). Shafi et al. (2021) used metabolomics to profile the changes in metabolite levels associated with mycorrhizal associations for nutrient uptake in *Glomus intraradices* and tomatoes (Chen et al., 2022). Untargeted metabolomics has identified lipid indicators of *Plasmopara viticola* inoculation in grapevines, whereas a study of maize genotypes interacting with nitrogen-fixing PGPB species revealed alterations in plant metabolites owing to bacterial nitrogen fixation (Diwan et al., 2022). Despite being underutilized in root-pathogen interaction studies compared to other omics methods, untargeted metabolomics provides valuable insights into plant-microbial interactions across various tissues, aiding the development of more effective crop and plant protection strategies (Demiwal et al., 2024). Detailed metabolomic analysis revealed *Pseudomonas syringae*-triggered hyperaccumulation of dihydrocamalexic acid (DHCA) in the apoplastic space of pad3 but not in cyp71a12/a13 plants, and infiltration of DHCA into cyp71a12/a13 mutant leaves restored resistance, indicating its role in restricting *P. syringae* growth in plants (Singh et al., 2023). Metabolomic profiling of rice infested with *Magnaporthe grisea* using liquid chromatography–mass spectrometry (LC-MS), gas chromatography-mass spectrometry (GC-MS), and nuclear magnetic resonance (NMR) methods revealed a varied metabolomic profile. Metabolomic profiling of *Dendrobium nobile* co-cultured with *Trichoderma longibrachiatum* using LC-MS revealed the metabolomic profile of dendrobine (Sarsaiya et al., 2024). Additionally, in maize, the resistance mechanism against *Fusarium graminearum* uncovered two metabolites, smiglaside and smilaside, whereas analysis of resistance to southern corn leaf blight identified polyphenols, lignin, and flavonoids through metabolite profiling using Fourier transform infrared (FT-IR) and NMR resonance (Manickam et al., 2023).

## 4 Modern web-based omics tools for plant-microbe interaction

**Figure 3:**
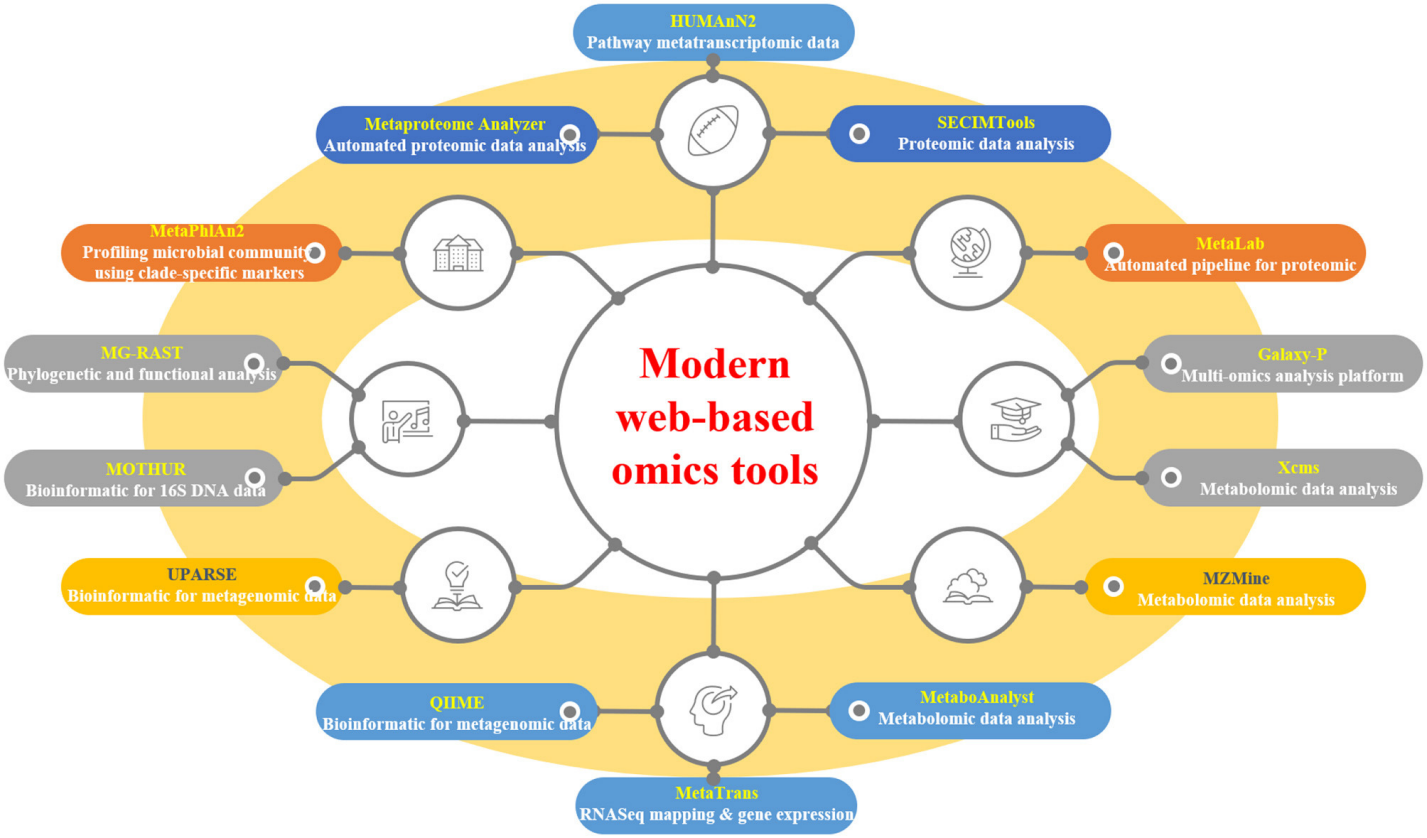
Selected modern web-based omics tools.

Web-based tools have revolutionized the analysis of modern “omics”-generated data, providing researchers with accessible and efficient platforms for data processing and interpretation. A plethora of tools cater to various omics disciplines, including automated pipelines, functional analysis, and pathway mapping (Table 3 and Figure 3). For proteomic analysis, tools like Metaproteome Analyzer (Schiebenhoefer et al., 2020), SECIMTools (Khan et al., 2019; Jibrin et al., 2021), and MetaLab (Starr et al., 2018) provide automated pipelines for proteomic data analysis, while Galaxy-P (Mehta et al., 2023) offers a multi-omics analysis platform. Metabolomic data analysis is facilitated by tools such as Xcms (Schweiger et al., 2014; Mueller et al., 2020), MZMine, and Metabolome MetaboAnalyst (Piasecka et al., 2019), which offer functionalities for pathway mapping and analysis. For metagenomic studies, tools such as QIIME, UPARSE, and MOTHUR aid in bioinformatics data analysis and operational taxonomic unit (OTU) generation (Lucaciu et al., 2019; Hupfauf et al., 2020), whereas MG-RAST and MetaPhlAn2 (Cassman et al., 2018; Dey and Ganguly, 2022) enable phylogenetic and functional analysis of metagenomic data. Metatranscriptomic analysis tools such as HUMAnN2, MetaTrans, and SAMSA (Liu et al., 2021) provide pathways for gene expression analysis and comprehensive pipelines for metatranscriptomic data analysis. Starr et al. (2018) introduced the Galaxy Integrated Omics (GIO) platform, aiming to streamline proteomics protein identification through genome/transcriptome-informed approaches. Galaxy-P (Galaxy for Proteomics) facilitates the integrative analysis of proteomic data alongside genomic or transcriptomic data. Its application in metaproteomics offers a comprehensive solution, encompassing database generation from sequencing data, iterative database searches, and subsequent taxonomic and functional analyses using external tools, such as Unipept and MEGAN (Shah et al., 2023). These web-based tools serve as invaluable resources for researchers to decipher complex biological datasets and to advance our understanding of omics-driven research across various disciplines.

**Table 3:** Modern web-based omics tools for plant-microbe interactions.

**Tool**	**Function**	**Advantages**	**References**
Metaproteome analyzer	Automated proteomic data analysis	Facilitates comprehensive analysis of proteomic data for understanding plant-microbe interactions	Schiebenhoefer et al., 2020
SECIMTools	Proteomic data analysis	Offers automated pipelines for efficient processing of proteomic data	Khan et al., 2019; Jibrin et al., 2021
MetaLab	Automated pipeline for proteomic analysis	Provides an efficient and user-friendly platform for proteomic data analysis	Starr et al., 2018
Galaxy-P	Multi-omics analysis platform	Integrates proteomic data with genomic/transcriptomic data for comprehensive analysis	Mehta et al., 2023
Xcms	Metabolomic data analysis	Offers functionalities for pathway mapping and functional analysis of metabolomic data	Schweiger et al., 2014; Mueller et al., 2020
MZMine	Metabolomic data analysis	Provides tools for processing and analyzing metabolomic data	Piasecka et al., 2019
MetaboAnalyst	Metabolomic data analysis	Enables functional analysis and pathway mapping of metabolomic data	Piasecka et al., 2019
QIIME	Bioinformatic analysis for metagenomic data	Facilitates bioinformatic analysis and OTU generation for metagenomic studies	Lucaciu et al., 2019
UPARSE	Bioinformatic analysis for metagenomic data	Enables efficient processing of metagenomic data for taxonomic analysis	Hupfauf et al., 2020
MOTHUR	Bioinformatic analysis for 16S DNA data	Provides tools for analyzing and visualizing 16S DNA data for microbial community analysis	Lucaciu et al., 2019; Hupfauf et al., 2020
MG-RAST	Phylogenetic and functional analysis of metagenomic data	Offers comprehensive analysis of metagenomic data, enabling both phylogenetic and functional insights	Cassman et al., 2018; Dey and Ganguly, 2022
MetaPhlAn2	Profiling microbial community using clade-specific markers	Facilitates the profiling of microbial communities and enables taxonomic analysis of metagenomic data	Cassman et al., 2018; Dey and Ganguly, 2022
HUMAnN2	Pathway analysis for metatranscriptomic data	Provides pathways for gene expression analysis and comprehensive pipelines for metatranscriptomic data analysis	Liu et al., 2021
MetaTrans	RNASeq mapping and gene expression analysis	Facilitates RNASeq mapping and analysis for gene expression studies	Liu et al., 2021
SAMSA	Complete analysis pipeline for metatranscriptomic data	Offers a comprehensive pipeline for metatranscriptomic data analysis	Liu et al., 2021
Galaxy-P	Integrated analysis of proteomics data with genomic/transcriptomic data	Streamlines proteomics protein identification through genome/transcriptome-informed approaches	Starr et al., 2018

## 5 Omics advancement and applications

**Figure 4:**
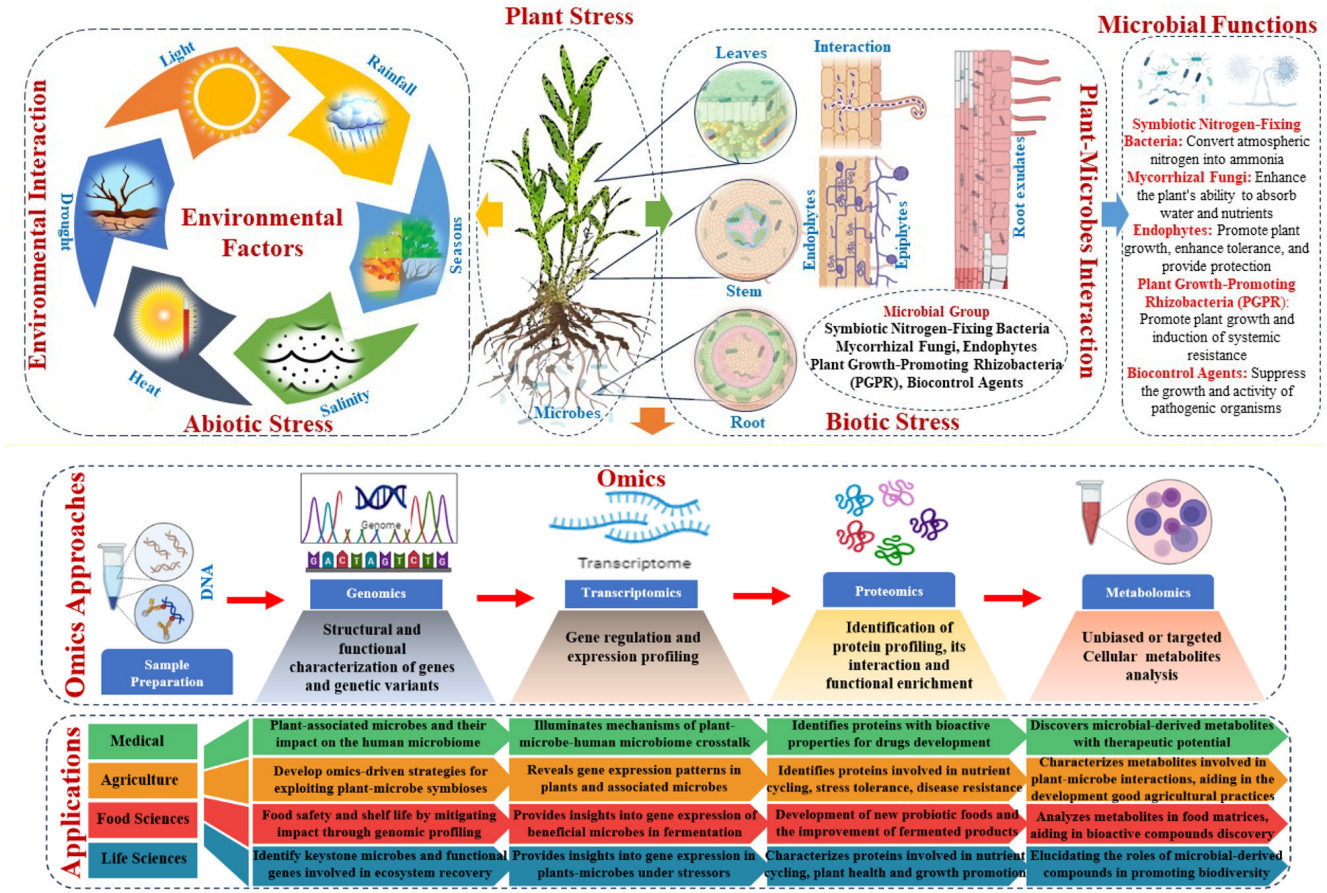
Omics approaches to understand host factors, microbial trait interactions, and their applications.

Genomic, transcriptomic, proteomic, and metabolomic technologies have transformed agriculture, medicine, food science, and life science. New insights from plant-microbe interaction omics illuminate the complex interactions between plants and microbes and their effects on crop productivity, sustainability, and environmental resilience (Sindelar, 2024). These advances have revealed a rich tapestry of plant-associated microbial species and their roles in nutrient cycling, disease control, stress tolerance, and plant-microbe signaling networks. The omics data revealed microbial traits that boosted plant growth, stress resistance, and nutrient uptake (Figure 4). Using these insights, omics-driven strategies have been developed to exploit beneficial plant-microbe symbioses, such as biofertilisation, pest management, and plant health and productivity. These interventions aim to improve soil fertility, chemical dependence, and agricultural sustainability (Ramlal et al., 2023). Precision agriculture uses plant-microbe interaction omics to optimize crop performance in diverse environments by fine-tuning microbial communities. Integrated with advanced computational tools, omics data enables predictive modeling, microbial consortia design, and agricultural management, boosting productivity and resilience (Shoaib et al., 2023).

Studies have shown that plant-associated microbes such as those present in the diet may affect the human microbiome, immune function, metabolism, and disease susceptibility. Omics-based approaches can illuminate the molecular mechanisms of plant-associated microbe-human microbiome crosstalk (Bashiardes et al., 2016). Researchers have identified microbial-derived metabolites, proteins, and signaling molecules that modulate host physiology and immune responses by characterizing the genomic, transcriptomic, proteomic, and metabolomic profiles of plants and human-associated microbes (Saravanakumar et al., 2022). Additionally, plant-microbe interaction omics may offer novel therapeutic approaches for human health. Plant-derived antimicrobial, anti-inflammatory, or immunomodulatory compounds can be used to develop drugs or dietary supplements for microbiome diseases or dysfunction (Mitropoulou et al., 2023). Some plant-associated microbes produce bioactive metabolites that kill human pathogens and promote the growth of commensal bacteria (Kandasamy and Kathirvel, 2023). Omics-based screening can identify these microbial-derived compounds and reveal their mechanisms of action, enabling the development of new antimicrobials or probiotics for infectious diseases, inflammatory disorders, and metabolic syndromes (Garia et al., 2024). Plant-microbe interaction omics can also help personalize medicine by revealing inter-individual microbial community variability and dietary component interactions (Wright et al., 2023; Speckmann et al., 2024).

Understanding and controlling foodborne pathogens and spoilage microorganisms are important. Researchers can identify potential pathogens and spoilage organisms, predict their behavior under different conditions, and develop strategies to mitigate their impact on food safety and shelf life by analyzing the genomic and proteomic profiles of microbial communities in food matrices. Using omics approaches, beneficial microbes used in fermentation processes, such as yogurt, cheese, and kimchi, can be studied to optimize fermentation conditions and develop new probiotic foods with improved nutritional and health benefits. In addition, plant-microbe interaction omics helps to create environmentally friendly food production methods (Joshi et al., 2023; Kumari et al., 2023). By understanding the role of plant-associated microbes in nutrient cycling, soil health, and plant growth promotion, researchers can identify strains that can be used as biofertilizers, biocontrol agents against plant pathogens, and biostimulants to boost crop productivity and resilience to environmental stressors. Microbial alternatives to conventional agrochemicals can reduce pollution and promote eco-friendly agriculture (Hussain et al., 2023; Rai et al., 2023).

Ecological restoration and conservation biology are increasingly being applied in the life sciences. Plant-microbe interaction omics allows researchers to study how habitat degradation, climate change, and pollution affect plant-microbe interactions and ecosystem resilience (Ge et al., 2023; Nadarajah and Abdul Rahman, 2023). Scientists can develop targeted restoration strategies to promote beneficial plant-microbe associations, ecosystem stability, and biodiversity by identifying keystone microbial species and functional genes involved in ecosystem recovery (Jansson et al., 2023; Timmusk et al., 2023). Plant-microbe interaction omics also helps us understand plant and microbial evolution and adaptation (Sa, 2024). Researchers can determine the genetics of trait variation, speciation events, and host-microbe co-evolutionary dynamics by comparing plant and microbe genomic and transcriptomic data across environments and evolutionary time scales (Kwak and Hansen, 2023; Mandal et al., 2023). These findings illuminate the evolutionary forces that shape biodiversity patterns and species interactions, informing conservation and ecosystem management strategies in the face of global environmental change. Omics technologies continue to innovate and discover diverse fields, solve complex problems, and reshape our understanding of biological systems and their applications.

## 6 Challenges, limitation, and future perspectives

Intensive agricultural practices on limited land with reduced fertilizer and agrochemical inputs present a significant global challenge. Plants such as humans interact with a diverse range of microorganisms that can have both beneficial and harmful effects. Despite ongoing advancements in omics-based approaches, experimental and computational validation procedures lack standardized protocols (Diwan et al., 2022). Discovering complex microbial communities in diverse environments remains a significant challenge, compounded by the fact that only a small fraction of microbes has been thoroughly characterized to date. The primary obstacle lies in the selection of the most suitable technology and methods to effectively address specific problems. Additionally, limitations in available databases present another hurdle that must be overcome to advance microbial research comprehensively (Kumar et al., 2023). Despite these advancements, the development of analytical methods to integrate multiple datasets remains a challenge in the field. To address this, there is a growing need for more multi-omics studies that incorporate classical approaches, such as metabolomics, transcriptomics, proteomics, and metagenomics, and embrace new and emerging techniques, such as genomics, epigenomics, and lipidomics (Kimotho and Maina, 2024).

The complexity of these interactions, encompassing various signaling molecules, pathways, and regulatory networks, makes deciphering their dynamics and outcomes challenging. Additionally, elucidating the precise mechanisms and kinetics of molecular signal recognition, transduction, and response in different plant-microbe systems requires sophisticated experimental approaches and analytical techniques. Furthermore, the diversity of microbial species and their adaptation strategies add another layer of complexity, necessitating comprehensive studies across diverse microbial taxa and ecological niches. Integrating multi-omics data from genomics, transcriptomics, proteomics, and metabolomics poses computational and analytical challenges, including data integration, standardization, and interpretation. Metabolomics, transcriptomics, proteomics, and metagenomics are invaluable tools for deciphering the complexities of plant-microbe interactions, but they pose distinct challenges. Metabolomics applies to the analytical complexity of diverse metabolites, identification hurdles, and dynamic nature of metabolite levels. Transcriptomics faces issues with RNA stability, the complexity of regulatory networks, and the need for robust bioinformatics tools. Proteomics encounters challenges in dealing with the complexity of the proteome, sensitivity in detecting low-abundance proteins, and in ensuring quantitative accuracy. Metagenomics struggles with data integration, sample contamination, and functional annotation.

Developing modern web-based omics tools for studying plant-microbe interactions presents several challenges. First, ensuring the integration and compatibility of diverse omics data types, including genomics, transcriptomics, proteomics, metabolomics, and metagenomics, within a single platform is crucial. This requires addressing issues related to data standardization, normalization, and interoperability across omics datasets. Second, incorporating advanced analytical algorithms and computational methods to handle the complexity and volume of omics data while maintaining scalability and efficiency poses significant technical hurdles. Additionally, ensuring user-friendly interfaces and intuitive designs to cater to users with varying levels of computational expertise are essential for the widespread adoption and usability of these tools. Moreover, ensuring data privacy, security, and compliance with ethical standards while facilitating data sharing and collaboration among researchers remains a challenge. Addressing these challenges requires interdisciplinary collaboration among bioinformaticians, biologists, and web developers, along with continuous updates and improvements to keep pace with advancements in omics technology and research.

While omics techniques have revolutionized our understanding of plant-microbe interactions, they are not without limitations. One significant challenge is the complexity and variability of biological systems, which can lead to issues such as ambiguity in data interpretation and difficulty distinguishing causative factors from correlations. In addition, omics approaches often generate vast amounts of data, necessitating sophisticated computational tools and expertise in analysis and interpretation. Moreover, the dynamic nature of plant-microbe interactions across different environmental conditions and developmental stages poses challenges in capturing the full spectrum of interactions. Another limitation is the dependency of omics techniques on high-quality reference genomes and databases, which may be lacking for non-model organisms or poorly characterized microbial taxa. Furthermore, although omics techniques provide insights into molecular mechanisms, they may not fully capture the spatial and temporal dynamics of the interactions occurring in complex ecosystems. Ethical considerations related to data sharing, privacy, and potential misuse also need to be addressed to ensure the responsible application of omics technologies in plant-microbe interaction studies. Despite these limitations, continued advancements in omics methodologies and interdisciplinary collaborations hold promise for overcoming these challenges and further enhancing our understanding of plant-microbe interactions for sustainable agriculture and environmental stewardship.

Future perspectives for omics approaches in understanding the benefits of plant-microbe interactions hold immense promise for unraveling the intricate dynamics of these relationships. Utilizing genomics, transcriptomics, proteomics, metabolomics, metagenomics, and other omics tools offers opportunities to delve deeper into the molecular mechanisms underlying various interactions, from mutualistic symbiosis to pathogenicity. By integrating multi-omics data and leveraging modern web-based tools, researchers can gain comprehensive insights into the genetic, transcriptional, proteomic, and metabolic landscapes of plant-microbe associations. This holistic understanding will not only advance fundamental knowledge but also pave the way for innovative agricultural practices, including the development of tailored microbial inoculants for sustainable crop production, identification of novel biocontrol strategies, and enhancement of plant resilience to biotic and abiotic stresses. Moreover, elucidating microbial-mediated molecular mechanisms, such as phytohormone modulation, effector secretion, and nutrient acquisition, holds promise for engineering beneficial plant-microbe interactions to improve crop productivity and environmental sustainability. As omics technologies continue to evolve and become more accessible, their integration with traditional approaches will undoubtedly revolutionize our understanding of plant-microbe interactions and shape the future of agriculture and ecosystem management.

## 7 Conclusion

In conclusion, the integration of omics technologies, including genomics, transcriptomics, proteomics, and metabolomics, represents a revolutionary leap in unraveling the complexities of plant-microbe interactions. By navigating the intricate factors that shape these associations, this review highlights the indispensable role of omics approaches in elucidating molecular nuances. From genomic insights into genetic variations in the metagenomic revelation of microbial communities and functions, each omics facet adds depth to our understanding. Transcriptomics unveils gene expression dynamics, whereas proteomics and metabolomics have shed light on protein functions and metabolic landscapes. Despite persistent challenges, such as standardization and data integration, omics technologies hold immense promise for optimizing agricultural strategies and fortifying resilient plant-microbe alliances. Future research should prioritize enhancing omics technology resolution and throughput, addressing challenges in data integration, and fostering interdisciplinary collaborations. Moreover, exploring the dynamics of plant-microbe interactions under diverse environmental conditions and elucidating the role of epigenomics will be pivotal. Overall, continued advancements in omics technologies offer exciting opportunities to deepen our understanding and harness the benefits of interactions for sustainable agriculture, environmental preservation, and plant health.

## Author contributions

AJ: Conceptualization, Data curation, Formal analysis, Investigation, Methodology, Resources, Visualization, Writing—original draft, Writing—review & editing. SS: Data curation, Formal analysis, Funding acquisition, Investigation, Visualization, Writing—original draft, Writing—review & editing. QG: Funding acquisition, Project administration, Resources, Supervision, Validation, Writing—review & editing. QW: Funding acquisition, Project administration, Resources, Supervision, Validation, Writing—review & editing. JS: Conceptualization, Funding acquisition, Investigation, Methodology, Project administration, Resources, Supervision, Writing—review & editing. RS: Writing—review & editing, Resources, Visualization, Software.

## References

[B1] Adams A. K. Landry D. Sykes V. R. Rickman T. Cham A. Timling A.. (2024). Maize Kernel-associated metagenomes reveal potential microbe-microbe interactions that underlie fusarium ear rot disease. Phytobiomes J. 23:74. 10.1094/PBIOMES-07-23-0074-R

[B2] Ahlawat O. P. Yadav D. Walia N. Kashyap P. L. Sharma P. Tiwari R.. (2024). Root exudates and their significance in abiotic stress amelioration in plants: a review. J. Plant Growth Regul. 24:7. 10.1007/s00344-024-11237-7

[B3] Ahmed S. Khan M. S. S. Xue S. Islam F. Ikram A. U. Abdullah M.. (2024). A comprehensive overview of omics-based approaches to enhance biotic and abiotic stress tolerance in sweet potato. Hortic Res. 11:uhae014. 10.1093/hr/uhae014 38464477 PMC10923648

[B4] Ajijah N. Fiodor A. Pandey A. K. Rana A. Pranaw K. (2023). Plant Growth-Promoting Bacteria (PGPB) with biofilm-forming ability: a multifaceted agent for sustainable agriculture. Diversity 15:112. 10.3390/d15010112

[B5] Ali M. H. Mandal S. Ghorai M. Lal M. K. Tiwari R. K. Kumar M.. (2023). “Chapter 6 - Perspectives of omics and plant microbiome,” in Plant Biology, Sustainability and Climate Change, Genomics, Transcriptomics, Proteomics and Metabolomics of Crop Plants, eds. A. Husen and A. Ahmad (Academic Press), 131–144. 10.1016/B978-0-323-95989-6.00014-0

[B6] Alshareef S. A. (2024). Metabolic analysis of the CAZy class glycosyltransferases in rhizospheric soil fungiome of the plant species *Moringa oleifera*. Saudi J. Biol. Sci. 31:103956. 10.1016/j.sjbs.2024.103956 38404538 PMC10891331

[B7] Anderson J. C. (2023). Ill communication: host metabolites as virulence-regulating signals for plant-pathogenic bacteria. Annu. Rev. Phytopathol. 61, 49–71. 10.1146/annurev-phyto-021621-114026 37253693

[B8] Assis A. Oliveira E. Donate P. Giuliatti S. Nguyen C. Passos G. (2014). “What is the transcriptome and how it is evaluated?,” in Transcriptomics in Health and Disease, ed. G. Passos (Cham: Springer), 3–48. 10.1007/978-3-319-11985-4_1

[B9] Barka G. D. Castro I. S. L. Alves D. R. de Almeida D. P. Caixeta E. T. (2023). “Chapter 4 - The role of receptor-like kinases in fungal/microbial resistance in plants,” in Plant Receptor-Like Kinases, eds. S. K. Upadhyay and Shumayla (Academic Press), 63–85. 10.1016/B978-0-323-90594-7.00019-3

[B10] Bashiardes S. Zilberman-Schapira G. Elinav E. (2016). Use of metatranscriptomics in microbiome research. Bioinform. Biol. Insights 10:BBI.S34610. 10.4137/BBI.S34610 27127406 PMC4839964

[B11] Bhargava P. Khan M. Verma A. Singh A. Singh S. Vats S.. (2019). “Metagenomics as a tool to explore new insights from plant-microbe interface,” in Plant Microbe Interface, eds. A. Varma, S. Tripathi, and R. Prasad (Cham: Springer), 271–289. 10.1007/978-3-030-19831-2_12

[B12] Bhattacharyya A. Mavrodi O. Bhowmik N. Weller D. Thomashow L. Mavrodi D.. (2023). Bacterial biofilms as an essential component of rhizosphere plant-microbe interactions. Methods Microbiol. 53, 3–48. 10.1016/bs.mim.2023.05.006 38415193 PMC10898258

[B13] Boutsika A. Michailidis M. Ganopoulou M. Dalakouras A. Skodra C. Xanthopoulou A.. (2023). A wide foodomics approach coupled with metagenomics elucidates the environmental signature of potatoes. iScience 26:105917. 10.1016/j.isci.2022.105917 36691616 PMC9860355

[B14] Carezzano M. E. Paletti Rovey M. F. Cappellari L. d. R. Gallarato L. A. Bogino P. Oliva M. d. l. M.. (2023). Biofilm-forming ability of phytopathogenic bacteria: a review of its involvement in plant stress. Plants 12:2207. 10.3390/plants12112207 37299186 PMC10255566

[B15] Carper D. L. Appidi M. R. Mudbhari S. Shrestha H. K. Hettich R. L. Abraham P. E.. (2022). The promises, challenges, and opportunities of omics for studying the plant holobiont. Microorganisms 10:2013. 10.3390/microorganisms10102013 36296289 PMC9609723

[B16] Cassman N. A. Lourenço K. S. do Carmo J. B. Cantarella H. Kuramae E. E. (2018). Genome-resolved metagenomics of sugarcane vinasse bacteria. Biotechnol. Biofuels 11:48. 10.1186/s13068-018-1036-9 29483941 PMC5822648

[B17] Chalupowicz L. Mordukhovich G. Assoline N. Katsir L. Sela N. Bahar O.. (2023). Bacterial outer membrane vesicles induce a transcriptional shift in arabidopsis towards immune system activation leading to suppression of pathogen growth in planta. J. Extracell. Vesicles 12:12285. 10.1002/jev2.12285 36645092 PMC9841551

[B18] Chandok I. K. Afreen H. Afreen R. Haider S. Moharana D. P. Hussain T.. (2022). “Chapter 9 - Functional genomics tools for studying microbe-mediated stress tolerance in plants,” in Mitigation of Plant Abiotic Stress by Microorganisms, eds. G. Santoyo, A. Kumar, M. Aamir, and S. Uthandi (Academic Press), 175–204. 10.1016/B978-0-323-90568-8.00009-2

[B19] Chao H. Zhang S. Hu Y. Ni Q. Xin S. Zhao L.. (2024). Integrating omics databases for enhanced crop breeding. J. Integr. Bioinform. 20:12. 10.1515/jib-2023-0012 37486120 PMC10777369

[B20] Chen J. Y. Sang H. Chilvers M. I. Wu C. H. Chang H. X. (2024). Characterization of soybean chitinase genes induced by rhizobacteria involved in the defense against *Fusarium oxysporum*. Front. Plant Sci. 15:1341181. 10.3389/fpls.2024.1341181 38405589 PMC10884886

[B21] Chen X. L. Sun M. C. Chong S. L. Si J. P. Wu L. S. (2022). Transcriptomic and metabolomic approaches deepen our knowledge of plant-endophyte interactions. Front. Plant Sci. 12:700200. 10.3389/fpls.2021.700200 35154169 PMC8828500

[B22] Chiquito-Contreras C. J. Meza-Menchaca T. Guzmán-López O. Vásquez E. C. Ricaño-Rodríguez J. (2024). Molecular insights into plant-microbe interactions: a comprehensive review of key mechanisms. Front. Biosci. Elite 16:9. 10.31083/j.fbe1601009 38538528

[B23] Chouchani E. T. (2022). Logic and mechanisms of metabolite signalling. Nat. Rev. Endocrinol. 18, 71–72. 10.1038/s41574-021-00618-7 34893789 PMC9148415

[B24] Contreras-Cornejo H. A. Schmoll M. Esquivel-Ayala B. A. González-Esquivel C. E. Rocha-Ramírez V. Larsen J.. (2024). Mechanisms for plant growth promotion activated by *Trichoderma* in natural and managed terrestrial ecosystems. Microbiol. Res. 281:127621. 10.1016/j.micres.2024.127621 38295679

[B25] Crandall S. G. Gold K. M. Jiménez-Gasco M. d. M. Filgueiras C. C. Willett D. S. (2020). A multi-omics approach to solving problems in plant disease ecology. PLoS ONE 15:e0237975. 10.1371/journal.pone.0237975 32960892 PMC7508392

[B26] da Silva L. I. Pereira M. C. de Carvalho A. M. X. Buttrós V. H. Pasqual M. Dória J. (2023). Phosphorus-solubilizing microorganisms: a key to sustainable agriculture. Agriculture 13:462. 10.3390/agriculture13020462

[B27] Datta R. (2024). Enzymatic degradation of cellulose in soil: a review. Heliyon 10:e24022. 10.1016/j.heliyon.2024.e24022 38234915 PMC10792583

[B28] De Palma M. Scotti R. D'Agostino N. Zaccardelli M. Tucci M. (2022). Phyto-friendly soil bacteria and fungi provide beneficial outcomes in the host plant by differently modulating its responses through (in)direct mechanisms. Plants 11:2672. 10.3390/plants11202672 36297696 PMC9612229

[B29] De Ryck J. Van Damme P. Goormachtig S. (2023). From prediction to function: current practices and challenges towards the functional characterization of type III effectors. Front. Microbiol. 14:1113442. 10.3389/fmicb.2023.1113442 36846751 PMC9945535

[B30] De Sousa B. F. S. Domingo-Serrano L. Salinero-Lanzarote A. Palacios J. M. Rey L. (2023). The T6SS-dependent effector Re78 of *Rhizobium etli* Mim1 benefits bacterial competition. Biology 12:678. 10.3390/biology12050678 37237492 PMC10215855

[B31] Demiwal P. Tayade S. Yadav S. R. Sircar D. (2024). A metabolomics perspective on root-derived plant immunity and phytohormone interaction. Physiol. Plant. 176:14150. 10.1111/ppl.14150

[B32] Dey K. K. Ganguly S. (2022). “Plant-microbe interactions in the age of sequencing,” in Plant-Microbe Interactions: Harnessing Next-Generation Molecular Technologies for Sustainable Agriculture, eds. J. Sahu, A. Vaishnav, and H. B. Singh (CRC Press), 113–127. 10.1201/9781003171416

[B33] Dhiman N. Uthoff J. Scharf B. Kumar V. (2024). “Plant-microbe interaction to improve soil health,” in Advancements in Microbial Biotechnology for Soil Health. Microorganisms for Sustainability, eds. R. K. Bhatia and A. Walia (Singapore: Springer), 113–127. 10.1007/978-981-99-9482-3_10

[B34] Diwan D. Rashid M. d. M. Vaishnav A. (2022). Current understanding of plant-microbe interaction through the lenses of multi-omics approaches and their benefits in sustainable agriculture. Microbiol. Res. 265:127180. 10.1016/j.micres.2022.127180 36126490

[B35] Doddavarapu B. Lata C. Shah J. M. (2024). Epigenetic regulation influenced by soil microbiota and nutrients: paving road to epigenome editing in plants. Biochim. Biophys. Acta 1868:130580. 10.1016/j.bbagen.2024.130580 38325761

[B36] Dubey R. K. Tripathi V. Prabha R. Chaurasia R. Singh D. P. Rao C. S.. (2020). “Metatranscriptomics and metaproteomics for microbial communities profiling,” in Unravelling the Soil Microbiome. Springer Briefs in Environmental Science (Cham: Springer), 51–60. 10.1007/978-3-030-15516-2_5

[B37] Dunn M. F. Becerra-Rivera V. A. (2023). The biosynthesis and functions of polyamines in the interaction of plant growth-promoting rhizobacteria with plants. Plants 12:2671. 10.3390/plants12142671 37514285 PMC10385936

[B38] Dutta P. Mahanta M. Singh S. B. Thakuria D. Deb L. Kumari A.. (2023). Molecular interaction between plants and *Trichoderma* species against soil-borne plant pathogens. Front. Plant Sci. 14:1145715. 10.3389/fpls.2023.1145715 37255560 PMC10225716

[B39] Elfaky M. A. (2024). Unveiling the hidden language of bacteria: anti-quorum sensing strategies for gram-negative bacteria infection control. Arch. Microbiol. 206:124. 10.1007/s00203-024-03900-0 38409503

[B40] Gamalero E. Bona E. Glick B. R. (2022). Current techniques to study beneficial plant-microbe interactions. Microorganisms 10:1380. 10.3390/microorganisms10071380 35889099 PMC9317800

[B41] Garia P. Chaubey K. K. Rawat H. Sinha A. Sharma S. Goyal U.. (2024). “Microbial metabolites and recent advancement,” in Fourth Congress on Intelligent Systems. CIS 2023, Lecture Notes in Networks and Systems, eds. S. Kumar, K. Balachandran, J. H. Kim, J. C. Bansal (Singapore: Springer), 175–194. 10.1007/978-981-99-9037-5_14

[B42] Gasser M. Keller J. Fournier P. Pujic P. Normand P. Boubakri H.. (2023). Identification and evolution of nsLTPs in the root nodule nitrogen fixation clade and molecular response of Frankia to AgLTP24. Sci. Rep. 13:16020. 10.1038/s41598-023-41117-1 37749152 PMC10520049

[B43] Ge J. Li D. Ding J. Xiao X. Liang Y. (2023). Microbial coexistence in the rhizosphere and the promotion of plant stress resistance: a review. Environ. Res. 222:115298. 10.1016/j.envres.2023.115298 36642122

[B44] Ghosh A. Mehta A. Khan A. M. (2019). “Metagenomic analysis and its applications,” in Encyclopedia of Bioinformatics and Computational Biology, eds. S. Ranganathan, M. Gribskov, K. Nakai, and C. Schönbach (Academic Press), 184–193. 10.1016/B978-0-12-809633-8.20178-7

[B45] Gómez-Godínez L. J. Aguirre-Noyola J. L. Martínez-Romero E. Arteaga-Garibay R. I. Ireta-Moreno J. Ruvalcaba-Gómez J. M. A.. (2023). Look at plant-growth-promoting bacteria. Plants 12:1668. 10.3390/plants12081668 37111891 PMC10145503

[B46] Goyal R. K. Habtewold J. Z. (2023). Evaluation of legume-rhizobial symbiotic interactions beyond nitrogen fixation that help the host survival and diversification in hostile environments. Microorganisms 11:1454. 10.3390/microorganisms11061454 37374957 PMC10302611

[B47] Grundy E. B. Gresshoff P. M. Su H. Ferguson B. J. (2023). Legumes regulate symbiosis with rhizobia via their innate immune system. Int. J. Mol. Sci. 24:2800. 10.3390/ijms24032800 36769110 PMC9917363

[B48] Gupta G. Chauhan P. S. Jha P. N. Verma R. K. Singh S. Yadav V. K.. (2024). Secretory molecules from secretion systems fine-tune the host-beneficial bacteria (PGPRs) interaction. Front. Microbiol. 15:1355750. 10.3389/fmicb.2024.1355750 38468848 PMC10925705

[B49] Gupta S. Pandey S. Nandi S. P. Singh M. (2023). Modulation of ethylene and ROS-scavenging enzymes by multifarious plant growth-promoting endophytes in tomato (*Solanum lycopersicum*) plants to combat Xanthomonas -induced stress. Plant Physiol. Biochem. 202:107982. 10.1016/j.plaphy.2023.107982 37651951

[B50] Han S. Na L. Rongchao Z. Xiuqin H. Wenyu Z. Bo Z.. (2023). Study on signal transmission mechanism of arbuscular mycorrhizal hyphal network against root rot of *Salvia miltiorrhiza*. Sci. Rep. 13:16936. 10.1038/s41598-023-43278-5 37805532 PMC10560300

[B51] Hupfauf S. Etemadi M. Fernández-Delgado Juárez M. Gómez-Brandón M. Insam H. Podmirseg S. M.. (2020). CoMA—an intuitive and user-friendly pipeline for amplicon-sequencing data analysis. PLoS ONE 15:e0243241. 10.1371/journal.pone.0243241 33264369 PMC7710066

[B52] Hussain M. Zahra N. Lang T. Zain M. Raza M. Shakoor N.. (2023). Integrating nanotechnology with plant microbiome for next-generation crop health. Plant Physiol. Biochem. 196, 703–711. 10.1016/j.plaphy.2023.02.022 36809731

[B53] Idris Usman N. Muazu Wali M. (2024). “Nitrogen fixation by rhizobacterial nif mechanism: an advanced genetic perspective,” in Updates on Rhizobacteria, ed. M. Gull (IntechOpen), 113. 10.5772/intechopen.1004087

[B54] Ilangumaran G. Subramanian S. Smith D. L. (2024). Complete genome sequences of *Rhizobium* sp. strain SL42 and *Hydrogenophaga* sp. strain SL48, microsymbionts of *Amphicarpaea bracteata*. Front. Microb. 3:1309947. 10.3389/frmbi.2024.1309947 PMC12993520 41853516

[B55] Jain A. Singh H. B. Das S. (2021). Deciphering plant-microbe crosstalk through proteomics studies. Microbiol. Res. 242:126590. 10.1016/j.micres.2020.126590 33022544

[B56] Jalal A. Júnior E. F. Teixeira Filho M. C. M. (2024). Interaction of zinc mineral nutrition and plant growth-promoting bacteria in tropical agricultural systems: a review. Plants 13:571. 10.3390/plants13050571 38475420 PMC10935411

[B57] Jansson J. K. McClure R. Egbert R. G. (2023). Soil microbiome engineering for sustainability in a changing environment. Nat. Biotechnol. 41, 1716–1728. 10.1038/s41587-023-01932-3 37903921

[B58] Jeon J. Kim K. T. Choi J. Cheong K. Ko J. Choi G.. (2022). Alternative splicing diversifies the transcriptome and proteome of the rice blast fungus during host infection. RNA Biol. 19, 373–386. 10.1080/15476286.2022.2043040 35311472 PMC8942408

[B59] Jiang C. Li Z. Zheng L. Yu Y. Niu D. (2023). Small RNAs: efficient and miraculous effectors that play key roles in plant-microbe interactions. Mol. Plant Pathol. 24, 999–1013. 10.1111/mpp.13329 37026481 PMC10346379

[B60] Jibrin M. O. Liu Q. Guingab-Cagmat J. Jones J. B. Garrett T. J. Zhang S.. (2021). Metabolomics insights into chemical convergence in *Xanthomonas perforans* and metabolic changes following treatment with the small molecule carvacrol. Metabolites 11:879. 10.3390/metabo11120879 34940636 PMC8706651

[B61] Joshi N. Ruparelia J. A. Saraf M. Jha C.K. (2023). “Techniques to study plant-microbe interactions that lead to efficient sustainable agriculture,” in Plant Microbiome for Plant Productivity and Sustainable Agriculture, eds. S. Chhabra, R. Prasad, N. R. Maddela, and N. Tuteja (Singapore: Springer), 401–421. 10.1007/978-981-19-5029-2_17

[B62] Joubert P. M. Krasileva K. V. (2024). Distinct genomic contexts predict gene presence-absence variation in different pathotypes of *Magnaporthe oryzae*. Genetics 2024:iyae012. 10.1093/genetics/iyae012 38290434 PMC10990425

[B63] Kalita P. Mohapatra B. Maruthi M. (2024). “Role of effectors in plant-pathogen interactions,” in Biotechnological Advances for Disease Tolerance in Plants, eds. K. Singh, R. Kaur, and R. Deshmukh (Singapore: Springer), 363–376. 10.1007/978-981-99-8874-7_15

[B64] Kandasamy G. D. Kathirvel P. (2023). Insights into bacterial endophytic diversity and isolation with a focus on their potential applications—a review. Microbiol. Res. 266:127256. 10.1016/j.micres.2022.127256 36410317

[B65] Kaya C. (2024). Microbial modulation of hormone signaling, proteomic dynamics, and metabolomics in plant drought adaptation. Food Energy Secur. 13:513. 10.1002/fes3.513

[B66] Khan N. Bano A. Babar M. A. (2019). Metabolic and physiological changes induced by plant growth regulators and plant growth promoting rhizobacteria and their impact on drought tolerance in *Cicer arietinum* L. PLoS ONE 14:e0213040. 10.1371/journal.pone.0213040 30830939 PMC6398973

[B67] Khatabi B. Gharechahi J. Ghaffari M. R. Liu D. Haynes P. A. McKay M. J.. (2019). Plant-microbe symbiosis: what has proteomics taught us? Proteomics 19:201800105. 10.1002/pmic.201800105 31218790

[B68] Khoshru B. Mitra D. Joshi K. Adhikari P. Rion M. S. I. Fadiji A. E.. (2023). Decrypting the multi-functional biological activators and inducers of defense responses against biotic stresses in plants. Heliyon 9:e13825. 10.1016/j.heliyon.2023.e13825 36873502 PMC9981932

[B69] Kimotho R. N. Maina S. (2024). Unraveling plant-microbe interactions: can integrated omics approaches offer concrete answers? J. Exp. Bot. 75, 1289–1313. 10.1093/jxb/erad448 37950741 PMC10901211

[B70] Koshila Ravi R. Muthukumar T. (2024). “Root exudates and their importance in arbuscular mycorrhizal symbiosis and nutrients navigation from inaccessible soil: an efficient mediator of mineral acquisition in nutrient deprived soil,” in Mycorrhizal Symbiosis and Agroecosystem Restoration, eds. R. A. Ansari, R. Rizvi, and I. Mahmood (Singapore: Springer), 101–123. 10.1007/978-981-99-5030-0_5

[B71] Kumar G. C. Chaudhary J. Meena L. K. Meena A. L. Kumar A. (2021). “Function-driven microbial genomics for ecofriendly agriculture,” in Microbes in Land Use Change Management, eds. J. S. Singh, S. Tiwari, C. Singh, and A. K. Singh (Elsevier), 389–431. 10.1016/B978-0-12-824448-7.00021-8

[B72] Kumar U. Raj S. Sreenikethanam A. Maddheshiya R. Kumari S. Han S.. (2023). Multi-omics approaches in plant-microbe interactions hold enormous promise for sustainable agriculture. Agronomy 13:1804. 10.3390/agronomy13071804

[B73] Kumari A. Kumari A. Sharma H. Sharma P. Bhattacharya S. Mishra T.. (2023). “Modern approaches in studying the role of plant-microbial interactions: a way towards the development of sustainable agriculture,” in New Frontiers in Plant-Environment Interactions. Environmental Science and Engineering, ed. T. Aftab (Cham: Springer), 69–91. 10.1007/978-3-031-43729-8_4

[B74] Kumari N. Kumawat K. C. (2024). “Chapter 19 - Microbial ACC-deaminase properties, functions and perspectives in climate stressed agriculture,” in Microbiome Research in Plants and Soil, Microbiome Drivers of Ecosystem Function, eds. J. A. Parray, N. Shameem, and D. Egamberdieva (Academic Press), 431–446. 10.1016/B978-0-443-19121-3.00008-9

[B75] Kwak Y. Hansen A. K. (2023). Unveiling metabolic integration in psyllids and their nutritional endosymbionts through comparative transcriptomics analysis. iScience 26:107930. 10.1016/j.isci.2023.107930 37810228 PMC10558732

[B76] Larekeng S. H. Ngadiman N. Khairina Y. Simarmata R. Christita M. (2024). Unraveling the potential of ACC Deaminase-producing microbes in various agricultural stresses: current status, limitations, and recommendations. Pak. J. Bot. 56:34. 10.30848/PJB2024-2(34)

[B77] Lee B. Lee J. I. Kwon S. K. Ryu C. M. Kim J. F. A. (2023). Marine bacterium with animal-pathogen-like type III secretion elicits the nonhost hypersensitive response in a land plant. Plant Pathol. J. 39, 584–591. 10.5423/PPJ.FT.09.2023.0125 38081318 PMC10721388

[B78] Liu Z. Ma A. Mathé E. Merling M. Ma Q. Liu B.. (2021). Network analyses in microbiome based on high-throughput multi-omics data. Brief Bioinform. 22, 1639–1655. 10.1093/bib/bbaa005 32047891 PMC7986608

[B79] Lucaciu R. Pelikan C. Gerner S. M. Zioutis C. Köstlbacher S. Marx H.. (2019). A bioinformatics guide to plant microbiome analysis. Front. Plant Sci. 10:1313. 10.3389/fpls.2019.01313 31708944 PMC6819368

[B80] Mahapatra R. Mishra P. Patel Z. M. (2023). “Chapter 9 - The molecular architecture of rhizobium-plant symbiosis in nitrogen fixation,” in The Chemical Dialogue Between Plants and Beneficial Microorganisms, eds. V. Sharma, R. Salwan, E. Moliszewska, D. Ruano-Rosa, and M. Jȩdryczka (Academic Press), 137–144. 10.1016/B978-0-323-91734-6.00006-5

[B81] Majdura J. Jankiewicz U. Gałazka A. Orzechowski S. (2023). The role of quorum sensing molecules in bacterial-plant interactions. Metabolites 13:114. 10.3390/metabo13010114 36677039 PMC9863971

[B82] Mandal M. Das S. Roy A. Rakwal R. Jones O. A. H. Popek R.. (2023). Interactive relations between plants, the phyllosphere microbial community, and particulate matter pollution. Sci. Tot. Environ. 890:164352. 10.1016/j.scitotenv.2023.164352 37230354

[B83] Manickam S. Rajagopalan V. R. Kambale R. Rajasekaran R. Kanagarajan S. Muthurajan R.. (2023). Plant metabolomics: current initiatives and future prospects. Curr. Iss. Mol. Biol. 45, 8894–8906. 10.3390/cimb45110558 37998735 PMC10670879

[B84] Manoharan B. Narayanasamy S. Joshi J. B. Jegadeesan S. Qi S. Dai Z.. (2023). “Molecular events and defence mechanism against biotic stress induced by bio-priming of beneficial microbes,” in Microbial Biocontrol: Molecular Perspective in Plant Disease Management. Microorganisms for Sustainability, eds. K. K. Bastas, A. Kumar, and U. Sivakumar (Singapore: Springer), 61–87. 10.1007/978-981-99-3947-3_3

[B85] Maphosa S. Moleleki L. N. Motaung T. E. (2023). Bacterial secretion system functions: evidence of interactions and downstream implications. Microbiology 169:1326. 10.1099/mic.0.001326 37083586 PMC10202321

[B86] Masenya K. Manganyi M. C. Dikobe T. B. (2024). Exploring cereal metagenomics: unravelling microbial communities for improved food security. Microorganisms 12:510. 10.3390/microorganisms12030510 38543562 PMC10974370

[B87] Meena M. Mehta T. Nagda A. Yadav G. Sonigra P. (2023). “Chapter 11 - PGPR-mediated synthesis and alteration of different secondary metabolites during plant-microbe interactions,” in Plant-Microbe Interaction - Recent Advances in Molecular and Biochemical Approaches, eds. P. Swapnil, M. Meena, Harish, A. Marwal, S. Vijayalakshmi, A. Zehra (Academic Press), 229–255. 10.1016/B978-0-323-91875-6.00002-5

[B88] Mehta S. Bernt M. Chambers M. Fahrner M. Föll M. C. Gruening B.. (2023). A Galaxy of informatics resources for MS-based proteomics. Expert Rev. Proteom. 20, 251–266. 10.1080/14789450.2023.2265062 37787106

[B89] Mishra A. K. Sudalaimuthuasari N. Hazzouri K. M. Saeed E. E. Shah I. Amiri K. M. A.. (2022). Tapping into plant-microbiome interactions through the lens of multi-omics techniques. Cells 11:3254. 10.3390/cells11203254 36291121 PMC9600287

[B90] Mitropoulou G. Stavropoulou E. Vaou N. Tsakris Z. Voidarou C. Tsiotsias A.. (2023). Insights into antimicrobial and anti-inflammatory applications of plant bioactive compounds. Microorganisms 11:1156. 10.3390/microorganisms11051156 37317131 PMC10222085

[B91] Mueller L. O. Borstein S. R. Tague E. D. Dearth S. P. Castro H. F. Campagna S. R.. (2020). Populations of *Populus angustifolia* have evolved distinct metabolic profiles that influence their surrounding soil. Plant Soil 448, 399–411. 10.1007/s11104-019-04405-2

[B92] Nadarajah K. Abdul Rahman N. S. N. (2023). The microbial connection to sustainable agriculture. Plants 12:2307. 10.3390/plants12122307 37375932 PMC10303550

[B93] Niazi P. Monib A. W. Ozturk H. Mansoor M. Azizi A. Hassand M. H.. (2023). Review on surface elements and bacterial biofilms in plant-bacterial associations. J. Res. Appl. Sci. Biotechnol. 2, 204–214. 10.55544/jrasb.2.1.30

[B94] Olanrewaju O. S. Glick B. R. Babalola O. O. (2024). Metabolomics-guided utilization of beneficial microbes for climate-resilient crops. Curr. Opin. Chem. Biol. 79:102427. 10.1016/j.cbpa.2024.102427 38290195

[B95] Orozco-Mosqueda M. d. C. Fadiji A. E. Babalola O. O. Santoyo G. (2023). Bacterial elicitors of the plant immune system: an overview and the way forward. Plant Stress 7:100138. 10.1016/j.stress.2023.100138

[B96] Pandey S. Blache A. Achouak W. (2024). Insights into bacterial extracellular vesicle biogenesis, functions, and implications in plant-microbe interactions. Microorganisms 12:532. 10.3390/microorganisms12030532 38543583 PMC10975234

[B97] Pantigoso H. A. Manter D. K. Fonte S. J. Vivanco J. M. (2023). Root exudate-derived compounds stimulate the phosphorus solubilizing ability of bacteria. Sci. Rep. 13:4050. 10.1038/s41598-023-30915-2 36899103 PMC10006420

[B98] Paul S. Parvez S. S. Goswami A. Banik A. (2024). Exopolysaccharides from agriculturally important microorganisms: conferring soil nutrient status and plant health. Int. J. Biol. Macromol. 262:129954. 10.1016/j.ijbiomac.2024.129954 38336329

[B99] Piasecka A. Kachlicki P. Stobiecki M. (2019). Analytical methods for detection of plant metabolomes changes in response to biotic and abiotic stresses. Int. J. Mol. Sci. 20:379. 10.3390/ijms20020379 30658398 PMC6358739

[B100] Priya P. Aneesh B. Harikrishnan K. (2021). Genomics as a potential tool to unravel the rhizosphere microbiome interactions on plant health. J. Microbiol. Methods 185:106215. 10.1016/j.mimet.2021.106215 33839214

[B101] Puranik S. Bundela V. Shylla A. Elakkya M. Shukla L. Singh S. K.. (2023). “Chapter 10 - Peeking into plant-microbe interactions during plant defense,” in Plant-Microbe Interaction - Recent Advances in Molecular and Biochemical Approaches, eds. P. Swapnil, M. Meena, Harish, A. Marwal, S. Vijayalakshmi, and A. Zehra (Academic Press), 167–200. 10.1016/B978-0-323-91876-3.00012-9

[B102] Rai S. Omar A. F. Rehan M. Al-Turki A. Sagar A. Ilyas N.. (2023). Crop microbiome: their role and advances in molecular and omic techniques for the sustenance of agriculture. Planta 257:27. 10.1007/s00425-022-04052-5 36583789

[B103] Ramlal A. Rani A. Nautiyal A. Kalra C. Kumari R. Kumar J.. (2023). Importance of omics approaches in plant-microbe interaction for plant disease control. Physiol. Mol. Plant Pathol. 128:102153. 10.1016/j.pmpp.2023.10215328553296

[B104] Rane N. R. Tapase S. Kanojia A. Watharkar A. Salama E. S. Jang M.. (2022). Molecular insights into plant-microbe interactions for sustainable remediation of contaminated environment. Bioresour. Technol. 344:126246. 10.1016/j.biortech.2021.126246 34743992

[B105] Rani A. Rana A. Dhaka R. K. Singh A. P. Chahar M. Singh S.. (2023). Bacterial volatile organic compounds as biopesticides, growth promoters and plant-defense elicitors: current understanding and future scope. Biotechnol. Adv. 63:108078. 10.1016/j.biotechadv.2022.108078 36513315

[B106] Rathnasamy S. A. Gothandapani S. Chellamuthu S. Chakraborty A. Gurusamy D. Roy A.. (2023). “Omics technologies unravelling the plant-pathogen interaction and stress response,” in Genomics of Plant-Pathogen Interaction and the Stress Response, eds. A. Mani and S. Kushwaha (CRC Press), 74–110. 10.1201/9781003153481

[B107] Ravelo-Ortega G. Raya-González J. López-Bucio J. (2023). Compounds from rhizosphere microbes that promote plant growth. Curr. Opin. Plant Biol. 73:102336. 10.1016/j.pbi.2023.102336 36716513

[B108] Regalado J. Lundberg D. S. Deusch O. Kersten S. Karasov T. Poersch K.. (2020). Combining whole-genome shotgun sequencing and rRNA gene amplicon analyses to improve detection of microbe-microbe interaction networks in plant leaves. ISME J. 14, 2116–2130. 10.1038/s41396-020-0665-8 32405027 PMC7368051

[B109] Restrepo-Leal J. D. Belair M. Fischer J. Richet N. Fontaine F. Rémond C.. (2023). Differential carbohydrate-active enzymes and secondary metabolite production by the grapevine trunk pathogen *Neofusicoccum parvum* Bt-67 grown on host and non-host biomass. Mycologia 115, 579–601. 10.1080/00275514.2023.2216122 37358885

[B110] Sa T. (ed.). (2024). “Chapter 1 - Plant-microbe interactions for enhanced plant tolerance to stress,” in Beneficial Microbes for Sustainable Agriculture Under Stress Conditions (Academic Press), 1–24. 10.1016/B978-0-443-13193-6.00001-4

[B111] Saarenpää S. Shalev O. Ashkenazy H. Carlos V. Lundberg D. S. Weigel D.. (2023). Spatial metatranscriptomics resolves host-bacteria-fungi interactomes. Nat. Biotechnol. 23:2. 10.1038/s41587-023-01979-2 37985875 PMC11392817

[B112] Samantara K. Shiv A. de Sousa L. L. Sandhu K. S. Priyadarshini P. Mohapatra S. R.. (2021). A comprehensive review on epigenetic mechanisms and application of epigenetic modifications for crop improvement. Environ. Exp. Bot. 188:104479. 10.1016/j.envexpbot.2021.104479

[B113] Santra H. K. Banerjee D. (2024). “Chapter 5 - Microbial extracellular polymeric substance: function and role against environmental stress,” in Nanobiotechnology for Plant Protection, Bacterial Secondary Metabolites, eds. K. A. Abd-Elsalam and H. I. Mohamed (Elsevier), 83–106. 10.1016/B978-0-323-95251-4.00018-1

[B114] Saravanakumar K. Santosh S. S. Ahamed M. A. Sathiyaseelan A. Sultan G. Irfan N.. (2022). Bioinformatics strategies for studying the molecular mechanisms of fungal extracellular vesicles with a focus on infection and immune responses. Brief Bioinform. 23:bbac250. 10.1093/bib/bbac250 35794708

[B115] Sarim K. M. Srivastava R. Ramteke P. W. (2020). “Chapter 9 - Next-generation omics technologies for exploring complex metabolic regulation during plant-microbe interaction,” in Microbial Services in Restoration Ecology, eds. J. S. Singh and S. R. Vimal (Elsevier), 123–138. 10.1016/B978-0-12-819978-7.00009-9

[B116] Sarsaiya S. Jain A. Shu F. Yang M. Pu M. Jia Q.. (2024). Enhancing dendrobine production in *Dendrobium nobile* through mono-culturing of endophytic fungi, *Trichoderma longibrachiatum* (MD33) in a temporary immersion bioreactor system. Front. Plant Sci. 15:1302817. 10.3389/fpls.2024.1302817 38348269 PMC10859523

[B117] Sartori M. Ferrari E. M'Barek R. Philippidis G. Boysen-Urban K. Borrelli P.. (2024). Remaining loyal to our soil: a prospective integrated assessment of soil erosion on global food security. Ecol. Econ. 219:108103. 10.1016/j.ecolecon.2023.108103

[B118] Scaria S. S. Ravi L. (2023). “Chapter 22 - Symbiotic associations of Frankia in actinorhizal plants,” in Developments in Applied Microbiology and Biotechnology, Microbial Symbionts, ed. D. Dharumadurai (Academic Press), 397–416. 10.1016/B978-0-323-99334-0.00002-5

[B119] Schiebenhoefer H. Schallert K. Renard B. Y. Trappe K. Schmid E. Benndorf D.. (2020). A complete and flexible workflow for metaproteomics data analysis based on MetaProteomeAnalyzer and Prophane. Nat. Protoc. 15, 3212–3239. 10.1038/s41596-020-0368-7 32859984

[B120] Schweiger R. Baier M. C. Müller C. (2014). Arbuscular mycorrhiza-induced shifts in foliar metabolism and photosynthesis mirror the developmental stage of the symbiosis and are only partly driven by improved phosphate uptake. Mol. Plant-Microbe Interact. 27, 1403–1412. 10.1094/MPMI-05-14-0126-R 25162317

[B121] Selwal N. Wani A. K. Akhtar N. Kaur M. Jassal P. S. (2023). Molecular insights of strigolactone biosynthesis, signalling pathways, regulatory roles, and hormonal crosstalks in plant systems. South Afri. J. Bot. 160, 9–22. 10.1016/j.sajb.2023.06.046

[B122] Sengupta K. Pal S. (2021). “Rhizospheric plant-microbe interactions releasing antioxidants and phytostimulating compounds in polluted agroecosystems,” in Antioxidants in Plant-Microbe Interaction, eds. H. B. Singh, A. Vaishnav, and R. Sayyed (Singapore: Springer), 157–179. 10.1007/978-981-16-1350-0_8

[B123] Shafi A. Zahoor I. Habib H. (2021). “Omics technologies to unravel plant-microbe interactions,” in Plant-Microbe Dynamics: Recent Advances for Sustainable Agriculture, 1st Edn, eds. T. B. Pirzadah, B. Malik, and K. R. Hakeem (CRC Press), 201–220. 10.1201/9781003106784

[B124] Shah K. Upadhye V. J. Shrivastav A. (2023). New Developments in Techniques Like Metagenomics and Metaproteomics for Isolation, Identification, and Characterization of Microbes from Varied Environment, 487–496.

[B125] Shah W. U. H. Lu Y. Liu J. Rehman A. Yasmeen R. (2024). The impact of climate change and production technology heterogeneity on China's agricultural total factor productivity and production efficiency. Sci. Tot. Environ. 907:168027. 10.1016/j.scitotenv.2023.168027 37898215

[B126] Sharma A. Choudhary P. Chakdar H. Shukla P. (2024). Molecular insights and omics-based understanding of plant-microbe interactions under drought stress. World J. Microbiol. Biotechnol. 40:42. 10.1007/s11274-023-03837-4 38105277

[B127] Shoaib M. Shah B. Sayed N. Ali F. Ullah R. Hussain I.. (2023). Deep learning for plant bioinformatics: an explainable gradient-based approach for disease detection. Front. Plant Sci. 14:1283235. 10.3389/fpls.2023.1283235 37900739 PMC10612337

[B128] Shumilina J. Soboleva A. Abakumov E. Shtark O. Y. Zhukov V. A. Frolov A.. (2023). Signaling in legume-rhizobia symbiosis. Int. J. Mol. Sci. 24:17397. 10.3390/ijms242417397 38139226 PMC10743482

[B129] Sindelar R. D. (2024). “Genomics, other “OMIC” technologies, precision medicine, and additional biotechnology-related techniques,” in Pharmaceutical Biotechnology, eds. D. J. A. Crommelin, R. D. Sindelar, and B. Meibohm (Cham: Springer International Publishing), 179–221. 10.1007/978-1-4614-6486-0_8

[B130] Singh G. Agrawal H. Bednarek P. (2023). Specialized metabolites as versatile tools in shaping plant-microbe associations. Mol. Plant 16, 122–144. 10.1016/j.molp.2022.12.006 36503863

[B131] Speckmann B. Ehring E. Hu J. Rodriguez Mateos A. (2024). Exploring substrate-microbe interactions: a metabiotic approach toward developing targeted synbiotic compositions. Gut Microbes 16:2305167. 10.1080/19490976.2024.2305716 38300741 PMC10841028

[B132] Starr A. E. Deeke S. A. Li L. Zhang X. Daoud R. Ryan J.. (2018). Proteomic and metaproteomic approaches to understand host-microbe interactions. Anal. Chem. 90, 86–109. 10.1021/acs.analchem.7b04340 29061041

[B133] Su G. Yu C. Liang S. Wang W. Wang H. (2024). Multi-omics in food safety and authenticity in terms of food components. Food Chem. 437:137943. 10.1016/j.foodchem.2023.137943 37948800

[B134] Swiatczak J. Kalwasińska A. Brzezinska M. S. (2024). Plant growth-promoting rhizobacteria: *Peribacillus frigoritolerans* 2RO30 and Pseudomonas sivasensis 2RO45 for their effect on canola growth under controlled as well as natural conditions. Front. Plant Sci. 14:1233237. 10.3389/fpls.2023.1233237 38259930 PMC10800854

[B135] Timmusk S. Pall T. Raz S. Fetsiukh A. Nevo E. (2023). The potential for plant growth-promoting bacteria to impact crop productivity in future agricultural systems is linked to understanding the principles of microbial ecology. Front. Microbiol. 14:1141862. 10.3389/fmicb.2023.1141862 37275175 PMC10235605

[B136] Tiwari P. Bose S. K. Park K. I. Dufossé L. Fouillaud M. (2024). Plant-microbe interactions under the extreme habitats and their potential applications. Microorganisms 12:448. 10.3390/microorganisms12030448 38543499 PMC10972407

[B137] Wahab A. Muhammad M. Munir A. Abdi G. Zaman W. Ayaz A.. (2023). Role of arbuscular mycorrhizal fungi in regulating growth, enhancing productivity, and potentially influencing ecosystems under abiotic and biotic stresses. Plants 12:3102. 10.3390/plants12173102 37687353 PMC10489935

[B138] Wangthaisong P. Piromyou P. Songwattana P. Wongdee J. Teamtaisong K. Tittabutr P.. (2023). The type IV secretion system (T4SS) mediates symbiosis between *Bradyrhizobium* sp. SUTN9-2 and legumes. Appl. Environ. Microbiol. 89:23. 10.1128/aem.00040-23 37255432 PMC10304904

[B139] Weidemüller P. Kholmatov M. Petsalaki E. Zaugg J. B. (2021). Transcription factors: bridge between cell signaling and gene regulation. Proteomics 21, 23–24. 10.1002/pmic.202000034 34314098

[B140] Weidenhamer J. D. Cipollini D. Morris K. Gurusinghe S. Weston L. A. (2023). Ecological realism and rigor in the study of plant-plant allelopathic interactions. Plant Soil 489, 1–39. 10.1007/s11104-023-06022-6

[B141] Wright A. T. Hudson L. A. Garcia W. L. (2023). Activity-based protein profiling—enabling phenotyping of host-associated and environmental microbiomes. Isr. J. Chem. 63:99. 10.1002/ijch.202200099

[B142] Wu D. Tian H. Xu F. Yang J. Feng W. Bell S.. (2024). The prodomain of *Arabidopsis metacaspase* 2 positively regulates immune signaling mediated by pattern-recognition receptors. New Phytol. 241, 430–443. 10.1111/nph.19365 37920109

[B143] Yin R. Cheng J. Lin J. (2024). The role of the type VI secretion system in the stress resistance of plant-associated bacteria. Stress Biol. 4:16. 10.1007/s44154-024-00151-3 38376647 PMC10879055

[B144] Yuan S. Ke D. Liu B. Zhang M. Li X. Chen H.. (2023). The Bax inhibitor GmBI-1α interacts with a Nod factor receptor and plays a dual role in the legume-rhizobia symbiosis. J. Exp. Bot. 74, 5820–5839. 10.1093/jxb/erad276 37470327

[B145] Zhang L. Chen F. Zeng Z. Xu M. Sun F. Yang L.. (2021). Advances in metagenomics and its application in environmental microorganisms. Front. Microbiol. 12:766364. 10.3389/fmicb.2021.766364 34975791 PMC8719654

[B146] Zhou D. Chen X. Chen X. Xia Y. Liu J. Zhou G.. (2023). Plant immune receptors interact with hemibiotrophic pathogens to activate plant immunity. Front. Microbiol. 14:1252039. 10.3389/fmicb.2023.1252039 37876778 PMC10591190

[B147] Zulfiqar M. Singh V. Steinbeck C. Sorokina M. (2024). Review on computer-assisted biosynthetic capacities elucidation to assess metabolic interactions and communication within microbial communities. Crit. Rev. Microbiol. 2024, 1–40. 10.1080/1040841X.2024.2306465 38270170

